# Determining the structure and properties of CO_2_ reduction photocatalysts: single atom cobalt atoms supported on various carbon nitrides†

**DOI:** 10.1039/d5ra03826j

**Published:** 2025-07-07

**Authors:** Qian Qian, N. Aaron Deskins

**Affiliations:** a Worcester Polytechnic Institute, Department of Chemical Engineering Worcester MA 01609 USA nadeskins@wpi.edu

## Abstract

Carbon nitride materials paired with Co atoms have been shown to be effective for CO_2_ photoreduction. However, the structures of Co/carbon nitrides are uncertain, especially when the degree of polymerization of the carbon nitrides is unknown. Literature has focused predominantly on the fully condensed carbon nitride, g-C_3_N_4_, despite evidence of other carbon nitrides being present in carbon nitride catalysts. We therefore used density functional theory (DFT) to model Co binding with molecular, partially condensed polymeric, and fully condensed polymeric carbon nitrides. We found strong binding of Co to the carbon nitrides, and larger coordination tended to lead to stronger binding of Co, as well as more cationic Co atoms. Co had a significant effect on photocatalytic processes. Co lowered band gaps significantly, enabling greater photoexcitation yields. Activation of CO_2_ into a bent, anionic state is an important initial step during CO_2_ reduction. Our calculations show that all Co/carbon nitride systems, except two-layer carbon nitrides, activated CO_2_. For bent CO_2_, strong interactions occurred between Co/carbon nitride systems, as evidenced by density of states plots and calculated interaction energies. On the other hand, weak interactions occurred with adsorbed linear CO_2_. Activation energies of CO_2_ spanned a wide range of values (−2.33 to −0.38 eV), and intermediate values over partially condensed carbon nitrides may be more likely to enable CO_2_ reduction. Our work provides insights and understanding of Co/carbon nitride catalysts, and motivates study of carbon nitrides beyond g-C_3_N_4_ as photocatalysts.

## Abstract 2

Carbon nitride materials paired with Co atoms have been shown to be effective for CO_2_ photoreduction.

## Graphical abstract

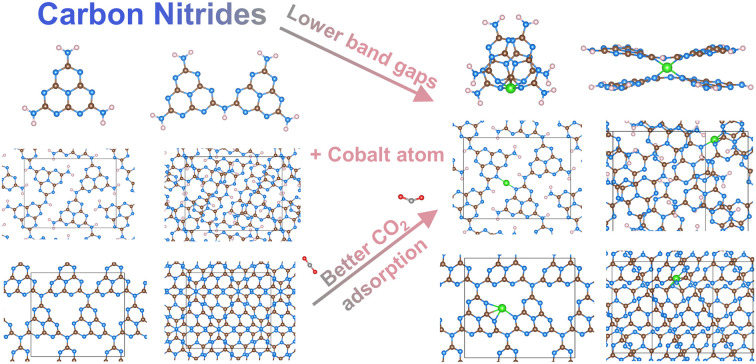

## 1 Introduction

Utilizing sunlight with efficient, low-cost photocatalysts is a vital step towards a clean, sustainable future. Carbon nitride materials have attracted much interest as photocatalysts,^1–9^ in part because these materials consist of cheap, abundant elements. They also have moderate band gaps for photoexcitation. These catalysts have been studied for a number of reactions, including water splitting,^10–14^ water purification,^15–17^ and CO_2_ reduction.^18–23^ Reducing atmospheric CO_2_ is an essential goal for mitigating climate change.

Carbon nitrides can have various compositions and structures.^24–28^ One of the most studied carbon nitrides is graphitic C_3_N_4_, or simply g-C_3_N_4_, which has a two-dimensional graphene-like structure with interconnected heptazine units. g-C_3_N_4_ may be considered a fully condensed two-dimensional material whereby melem molecules (C_6_N_10_H_6_) combine together to release NH_3_.^28–30^ Both experimental and computational work have shown that various polymeric carbon nitrides form due to incomplete condensation under different reaction conditions.^13,28,31–36^ Density functional theory (DFT) work suggests that g-C_3_N_4_ may be hard to synthesize under typical synthesis conditions (*e.g.*, atmospheric pressure), and that partially condensed polymeric carbon nitride structures (*e.g.*, melon – [C_6_N_9_H_3_]_*n*_) may be more prevalent than many researchers have considered.^31–33,35,37^ Furthermore, most experimental carbon nitrides contain a significant amount of hydrogen^25,28,35^ and also deviate from the ideal g-C_3_N_4_ C : N ratio of 0.75, ^24,34^ again indicating the likely presence of partially condensed polymers in many studies. Indeed recent DFT studies have attempted to more fully understand polymeric carbon nitrides such as melon.^31,38–40^ Other DFT work^33^ suggests that polymeric carbon nitrides may have many different structural domains (both partially and fully condensed) rather than a single structure. Thus, there is a need to better understand and characterize polymeric carbon nitrides beyond just two-dimensional g-C_3_N_4_, particularly partially condensed carbon nitrides. In this work we concentrate on characterizing polymeric carbon nitride materials as photocatalysts for CO_2_ reduction, especially when paired with single metal atoms.

One promising way to increase photocatalytic activity of carbon nitrides is to pair them with single metal atoms.^41–48^ Single atom catalysts (SACs) have attracted much interest because they can have high catalytic activity per atom, and can also have novel chemical or catalytic properties compared to bulk materials. The carbon nitride acts as a support and photoabsorber for these SACs. Synergy between the metal atoms and carbon nitride can decrease band gaps/increase photoabsorption,^49–55^ and also increase charge separation/lower charge recombination (necessary for long-lived charge carriers).^42,46,55–59^ Such carbon nitride/single metal atom catalysts may be active for reactions such as the H_2_ evolution reaction^52,58,60,61^ or CO_2_ reduction.^62–66^ Of particular interest is Co, which has demonstrated activity for CO_2_ photoreduction.^67–74^ Using Co/carbon nitride catalysts for CO_2_ photoreduction is a promising way to deal with the CO_2_ problem. Co/carbon nitride catalysts have demonstrated better adsorption of CO_2_, higher light utilization, and higher production of CO compared to pure carbon nitrides.^72^

Identifying the geometry of Co/carbon nitrides is an important step towards connecting their structure to catalytic activity, as well as a precursor for simulating these materials using atomistic modeling methods. Without details on their structure, detailed reaction mechanisms and structure–activity relationships are unclear. Indeed, there is ambiguity about the structure of Co/carbon nitrides. For example, the Co–N coordination number of Co/carbon nitride catalysts has been reported to be in the range of two to four depending on the material.^61,68,71–76^ We have shown through theory and experiment a potential four-coordinated structure.^74^ In contrast, Co may be four- or six-coordinated in bulk cobalt oxides. Other work involving doped carbon nitrides reported four-coordinated Co–P structures.^77^ Besides details on the geometry of Co atoms, identifying the oxidation state, effect on photoabsorption, and potential reaction sites are important for clarifying structure–activity relationships. The modeling literature has predominantly focused on how Co SACs bind to g-C_3_N_4_.^78–81^ A few DFT papers have been published on Co SACs supported by melon, but these papers assumed a dehydrogenated form of melon,^82,83^ while another paper modeled SACs to one-layer melon.^84^ Accordingly, it is critical to understand the nature of Co interacting with a full range of carbon nitrides, including molecular, partially condensed polymers, and fully condensed polymers, as well as multi-layer carbon nitrides, as all these types of carbon nitrides may be present in real-world catalysts.

In this work, we used DFT to model Co/carbon nitride catalysts in order to clarify their structures and determine potential photocatalytic activity, especially for CO_2_ photoreduction. We modeled molecular carbon nitrides, partially condensed carbon nitrides, and fully condensed two-dimensional carbon nitrides. We assessed how different carbon nitride supports interact with Co atoms, leading to different complexes and Co oxidation states. We also elucidated how Co atoms may affect band gaps of these materials, which is important for photocatalysis. Finally we modeled the activation of CO_2_ over these materials, a necessary first step towards CO_2_ photoreduction. Our work provides fundamental details on these catalysts, and ties together how different carbon nitride supports affect SAC stability, structure, and photocatalytic activity.

## 2 Methodology

### 2.1 Simulation parameters

We used the Vienna *Ab initio* Simulation Package (VASP)^85–88^ with the Perdew Burke Ernzerhof exchange-correlation functional^89^ for all the DFT simulations. Valence electrons were represented by a plane wave basis set, while core electrons were represented by projector augmented wave (PAW) potentials.^90,91^ The number of valence electrons for each atom were: Co (9), C (4), N (5), H (1), and O (6). A cutoff energy of 450 eV was used for the plane waves. Criterion for convergence of electronic and ionic relaxations were 1 × 10^−5^ eV and 0.02 eV Å^−1^. The Grimme D3 correction^92^ with Becke–Johnson damping^93^ was used to incorporate van der Waals interactions. We also used Gaussian smearing with a *σ* value of 0.1 eV. Charges of atoms were determined using Bader charge analysis.^94–96^ We used the HSE06 hybrid functional,^97–99^ combined with Gaussian smearing and a *σ* value of 0.01 eV, for calculating higher resolution band gaps and the density of states. The VASPKIT code was used for post-processing of our simulations.^100^ Images of structures were generated using VESTA.^101^

Binding energies (Δ*E*_binding_) of Co atoms were calculated by eqn (1):

1 Δ*E*_binding_ = *E*_Co/Carbon Nitride_ − *E*_Carbon Nitride_ − *E*_Co_

Here *E*_Co/Carbon Nitride_ is the energy of the Co/carbon nitride complex, *E*_Carbon Nitride_ is the energy of the carbon nitride material, and *E*_Co_ is the energy of a lone Co atom. Adsorption energies (Δ*E*_ads_) of CO_2_ were calculated by eqn (2):

2 Δ*E*_ads_ = *E*_CO_2_/Co/Carbon Nitride_ − *E*_Co/Carbon Nitride_ − *E*_CO_2__

Here *E*_CO_2_/Co/Carbon Nitride_ is the energy of the CO_2_/Co/carbon nitride complex and *E*_CO_2__ is the energy of a CO_2_ molecule.

### 2.2 Structural models

**Fig. 1:**
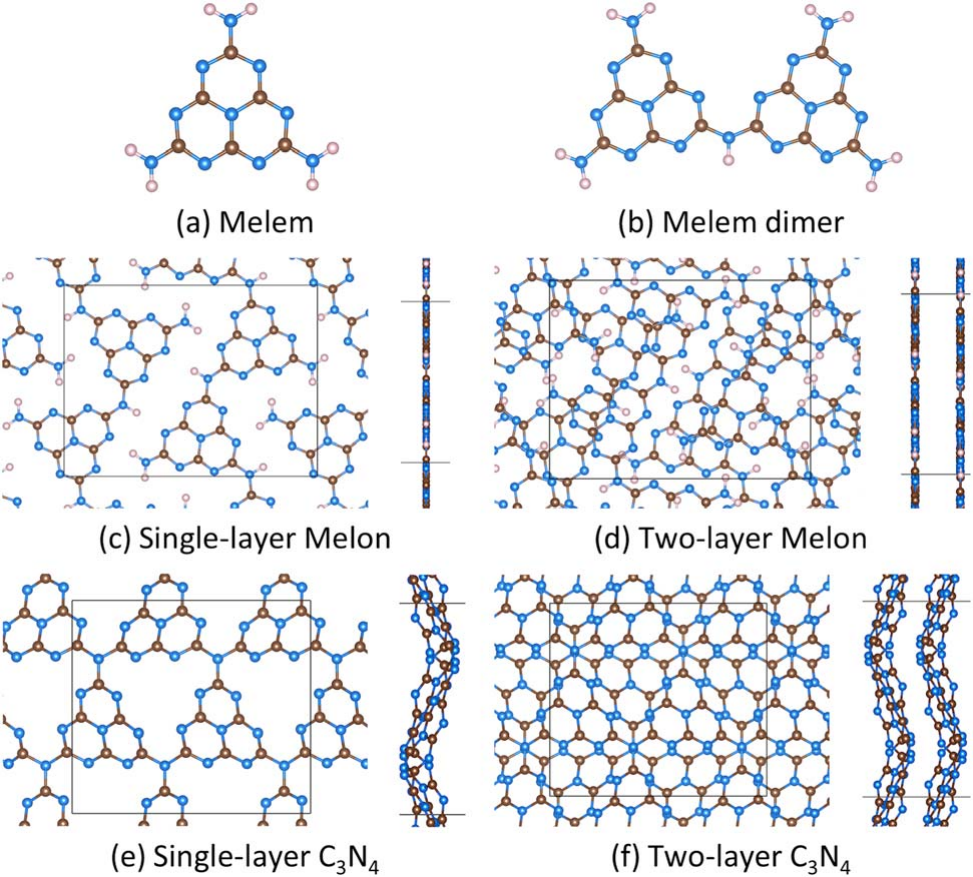
Carbon nitrides molecules, melem (a) and melem dimer (b), modeled in this work. Periodic carbon nitride polymers , melon (c and d) and graphitic C_3_N_4_ (e and f), modeled in this work. Also shown are the simulation cells for the periodic structures. Brown spheres represent carbon atoms, blue spheres represent nitrogen atoms, and pink spheres represent hydrogen atoms.

In this work we modeled several carbon nitrides, including polymeric building blocks (melem and melem dimer), partially condensed polymers (melon), and fully condensed carbon nitrides (g-C_3_N_4_). Fig. 1 shows the carbon nitrides we modeled. These carbon nitrides have been previously modeled.^31,32,40^ Interestingly, recent simulations suggest that experimentally relevant carbon nitrides consist of both partially and fully condensed polymer structures.^33^ Melem is a building block of polymeric carbon nitrides.^24,25,102^ Melem dimers may also form during the polymerization process. Melem and melem dimers were modeled in large supercells of size 30 Å × 30 Å × 30 Å. We used a single *k*-point for these calculations. Condensation of melem can form melon.^24,33,102^ Our melon model was based on Fina *et al.*^37^ and is further discussed in the ESI Section 1.† As Fig. 1 shows, the stable melon structure consisted of melon strands arranged together within the simulation cell. The single-layer melon structure had lattice lengths of 12.8 Å and 16.8 Å (based on lattice optimization) with a vacuum spacing of ∼30 Å, and included 72 atoms. Our two-layer melon structure was also based on Fina *et al.*’s melon structure.^37,40^ This simulation cell had lattice vectors of 12.8 Å and 16.7 Å after lattice optimization. The *z* lattice vector was set to ∼30 Å, resulting in a vacuum spacing of ∼27 Å.

Single-layer g-C_3_N_4_ (similar to Melissen *et al.*^31^) was modeled using a (2 × 2) orthorhombic supercell containing 56 atoms and with optimized lattice lengths of 11.8 Å and 13.1 Å. The vacuum space was ∼30 Å. Corrugated g-C_3_N_4_ is more stable than planar g-C_3_N_4_, as found in literature^31^ and our own calculations, so we modeled all g-C_3_N_4_ as corrugated. We also modeled two-layer g-C_3_N_4_, as layers of g-C_3_N_4_ may be attracted by van der Waal forces. Two-layer g-C_3_N_4_ (taken from Botari *et al.*^32^) was modeled with an orthorhombic cell containing 112 atoms with lattice parameters being 11.9 Å and 13.3 Å. The vacuum space was ∼26 Å. Test calculations (see Table S1†) indicated that a (2 × 2 × 1) *k*-point mesh was sufficient for all the periodic polymer calculations (*i.e.*, melon and g-C_3_N_4_), and we used such a *k*-point mesh in our work for all the periodic carbon nitrides. Analysis of our carbon nitride models (see the ESI Section 2†) indicates agreement with literature.

A challenge is quickly identifying whether Co–C or Co–N bonds occur in a structure, or the coordination number of Co. We used a simple procedure of designating a bond if the Co–X (X = C, N) bond distance was less than some threshold cutoff value, similar to previous work.^103,104^ Co–X bond distances in Co/carbon–nitrogen materials (such g-C_3_N_4_ and nitrogen doped graphene) were found to range from 2.02 to 2.44 Å (Co–C) and 2.04 to 2.37 Å (Co–N) (from both experimental^61,75,105–108^ and computational work^61,75,105^). Therefore, we set the cutoff value for determining Co–C and Co–N bonds to 2.5 Å.

## 3 Results and discussion

### 3.1 Binding of Co to carbon nitride materials

#### 3.1.1 Binding of Co to molecular carbon nitrides

**Fig. 2:**
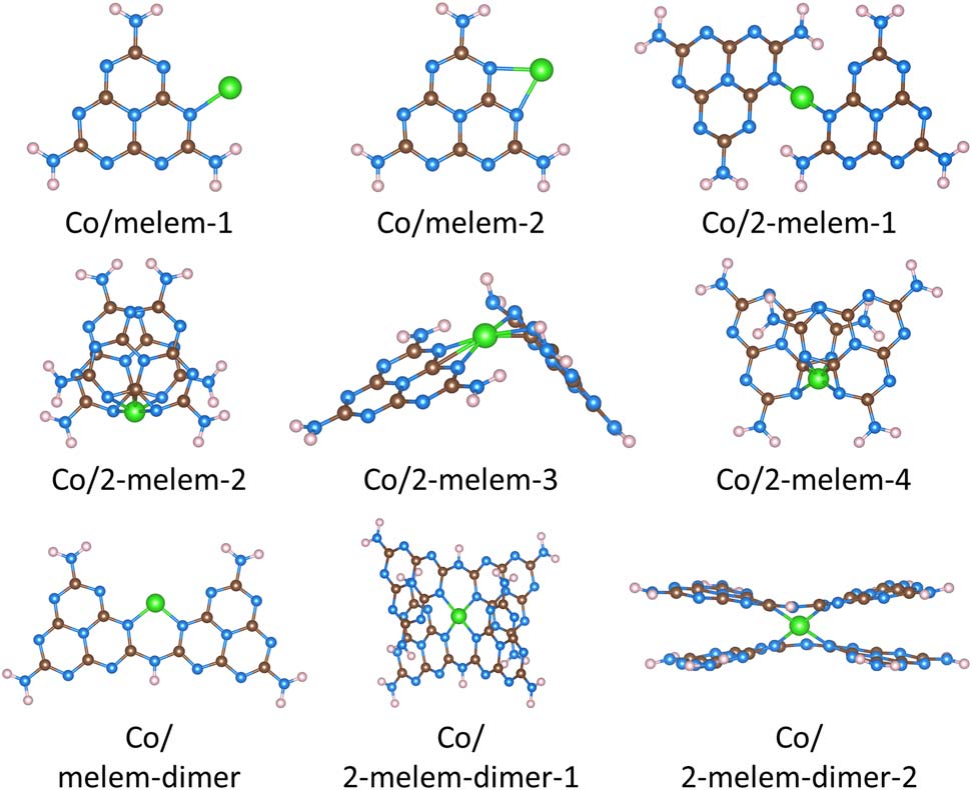
Stable structures where Co was bound to carbon nitride molecules. Brown spheres represent carbon atoms, blue spheres represent nitrogen atoms, pink spheres represent hydrogen atoms, and green spheres represent cobalt atoms. The notation system is decribed in the main text. When two molecules are present, these are designated by the prefix ’2-’. Each unique configuration is identified by the final number (*e.g.*, ’-1′, or ’-2′). A simplified representation of each structure showing only the local coordination around the Co atom is provided in Fig. S3.†

We considered how single Co atoms bind to the different carbon nitrides, first examining Co bound to carbon nitride molecules. In order to identify the most stable structures, we modeled several possible Co/molecular complexes. For example, we modeled Co binding to two adjacent N atoms within a single carbon nitride molecule, as well as combinations in which N atoms from two carbon nitride molecules interacted with the Co atom. Furthermore, the Co atom was placed in different orientations, such as the same plane with the carbon nitrides or sandwiched between two molecules. We present those structures which are most stable after geometry optimization, or lowest in energy, in Fig. 2. Table 1 also summarizes details on these structures. We note that several structures had quite different geometries, but similar binding energies for Co. Thus, we present several different structures for each molecule, and label them based on the number of carbon nitride molecules and their configuration. A simplified representation of each structure showing only the local coordination around the Co atom is provided in Fig. S3.† For example, Co/melem-1 refers to the first structure with Co binding with one melem, while Co/melem-2 refers to the second structure with Co binding with one melem. Co/2-melem-1 refers to the first configuration of Co binding with two melem molecules. Co/melem-dimer-1 refers to the first configuration with Co binding to a melem dimer, while Co/2-melem-dimer-1 refers to first configuration with Co binding to two melem dimers. In addition to the structures presented, we also identified other less stable structures, as well as structures that were similar to those in the main text. These alternative structures are included in Fig. S1.†

**Table 1:** Results for Co bound to molecular carbon nitrides. Shown are calculated Co Bader charges, Co binding energies, Co coordination numbers (Co–N coordination number, followed by Co–C coordination number), and distances between Co and closest atoms (typically N). In some cases Co was bound to C atoms, as indicated in the table. Structures in the table correspond to the same structures shown in Fig. 2. Simplified images of the local structures are found in Fig. S3

Models	Co charge |*e*^−^|	Δ*E*_binding−Co_ (eV)	Co CN	Co–M distance (Å)
Co/melem-1	0.10	−1.11	1	2.07
Co/melem-2	0.15	−1.12	2	2.16 2.16
Co/2-melem-1	0.34	−3.14	2	1.86 1.86
Co/2-melem-2	0.68	−3.36	4/2	2.02 2.17 2.15 2.02 1.90(C) 1.90(C)
Co/2-melem-3	0.66	−3.31	4/2	2.03 2.10 2.00 2.10 2.45(C) 1.88(C)
Co/2-melem-4	0.70	−3.15	4/2	2.20 2.02 2.04 2.16 1.90(C) 1.90(C)
Co/melem-dimer	0.55	−2.78	2	1.86 1.86
Co/2-melem-dimer-1	0.96	−5.23	4	1.96 2.01 1.96 2.01
Co/2-melem-dimer-2	0.77	−5.28	4	1.91 1.91 1.91 1.91

Binding energies for all molecular structures were found to be exothermic, ranging from −5.28 to −1.11 eV. The weakest binding occurred with just single melem molecules (*e.g.*, −1.11 eV for Co/melem-1), where the Co interacted with just one or two N atoms. Notably, binding energies were more exothermic when Co interacted with two molecules (*e.g.*, −3.36 eV for Co/2-melem-2), such as with two melem molecules or with two melem dimers. As noted above, Co binding with single melem molecules resulted in either one or two Co–N coordination. Co binding with other molecular carbon nitrides (such as multiple melems or dimers) typically resulted in larger coordination numbers (up to 6), as well as more exothermic binding energies (ranging from −5.28 to −2.78 eV). Notably, Co binding with two melems tended to form Co–C moieties, whereas no other carbon nitride molecules had such bonds.

Fig. S2† shows a comparison between binding energies and Co coordination number. *R*^2^ values for a regression fit are around 0.5, indicating reasonably moderate correlation. Our results show that Co with higher coordination was generably most stable, as seen for example by Co/2 melems or Co/2 melem dimers, where the most stable structures have Co with four Co–N coordination (Co/2-melem-2 and Co/2-melem-dimer-2). Coordination values beyond four tend to have a small affect on binding energies (see Fig. S2†). We also observed a variety of geometries for the Co/melem complexes. Simplified representations indicating the local geometries and coordination coordination around the Co atom are provided in Fig. S3.† The local geometries are also listed in Table S4.† Notably, the bent and linear geometries had the weakest binding energies (−1.12 eV for Co/melem-2, −2.78 eV for Co/melem-dimer, and −3.14 eV for Co/2-melem-1), while the strained trigonal prismatic, tetrahedral, and square planar geometries had the strongest binding energies, ranging from −5.28 to −3.15 eV, likely due to the increased number of Co–X bonds in these later structures.

#### 3.1.2 Binding of Co to partially condensed polymeric carbon nitrides

**Fig. 3:**
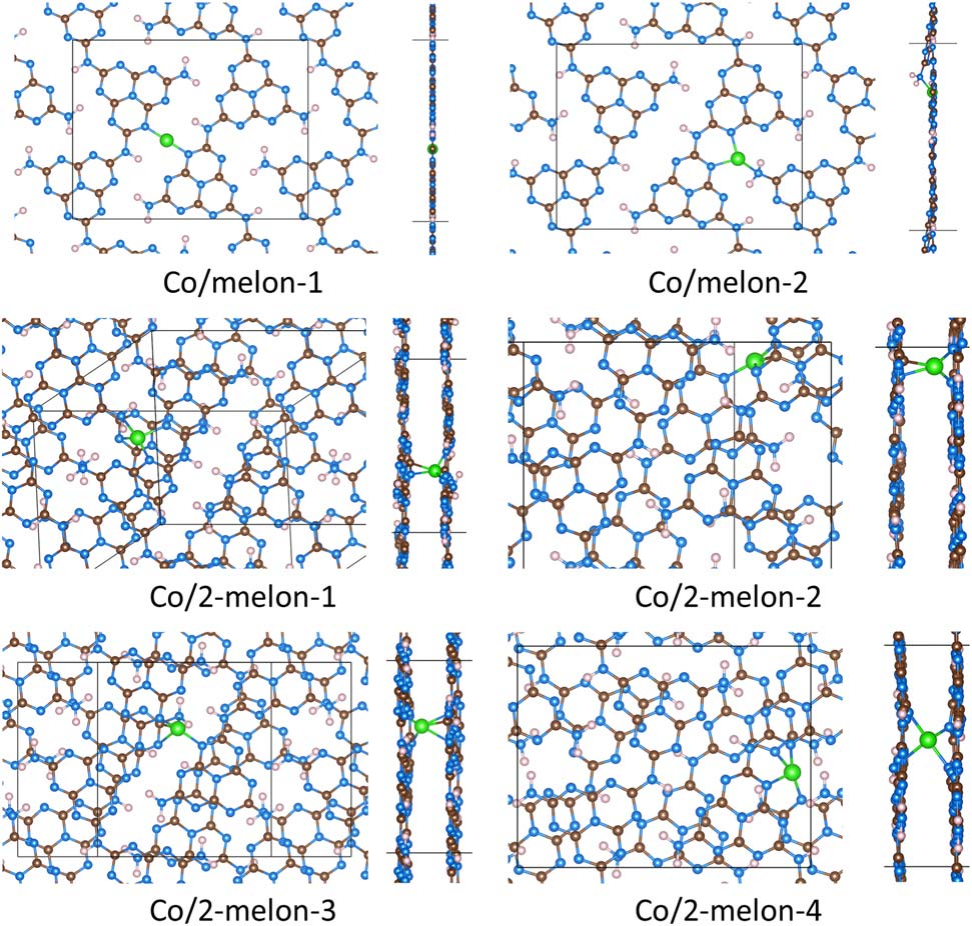
Stable structures where Co was bound to melon strands. Brown spheres represent carbon atoms, blue spheres represent nitrogen atoms, pink spheres represent hydrogen atoms, and green spheres represent cobalt atoms. When two melons are present, these are designated by the prefix ’2-’. Each unique configuration is identified by the final number (*e.g.*, ’-1′, ’-2′, *etc.*). Top and side views are shown. Simplified images of the Co/melon structures are found in Fig. S4.†

Fig. 3 shows structures for Co bound to a partially condensed polymeric carbon nitride, melon. Details of these calculations are summarized in Table 2. We present several different structures for Co binding to single-layer melon (denoted as Co/melon) and two-layer melon (denoted as Co/2-melon), with each configuration designated by a different number (*e.g.*, 1, 2, *etc.*). The most stable structure with Co binding to single-layer melon was Co/melon-1 (*E*_binding_ = −3.00 eV), where Co was coordinated to two N atoms. An alternative structure (Co/melon-2) had a binding energy of −2.84 eV, and Co was coordinated to three N atoms. Simplified images of Co/melon structures illustrating the local geometries are found in Fig. S4.† In Co/melon-1, Co was bound to nitrogen atoms from two melon strands, forming a linear structure. Co/melon-2 formed a trigonal planar structure interacting with two strands of melon.

**Table 2:** Results for Co bound to carbon nitride polymers. Shown are calculated Co Bader charges, Co binding energies, Co coordination numbers (Co–N coordination number, followed by Co–C coordination number), and distances between Co and closest atoms (typically N). In some cases Co was bound to C atoms, as indicated in the table. Structures in the table correspond to the same structures shown in Fig. 3 and 4. Simplified images of the local structures are found in Fig. S4

Models	Co charge |*e*^−^|	Δ*E*_binding−Co_ (eV)	Co CN	Co–M distance (Å)
Co/melon-1	0.49	−3.00	2	1.83 1.83
Co/melon-2	0.72	−2.84	3	2.06 1.90 1.92
Co/2-melon-1	0.78	−3.36	4/1	1.96 2.30 1.94 2.01 1.99(C)
Co/2-melon-2	0.77	−3.61	3/1	1.99 1.97 1.93 1.94(C)
Co/2-melon-3	0.78	−3.46	4/1	1.94 2.02 2.36 1.98 1.96(C)
Co/2-melon-4	0.86	−3.35	4	2.02 2.16 2.01 2.06
Co/C_3_N_4_-1	0.71	−2.88	2/1	1.87 1.89 1.93(C)
Co/C_3_N_4_-2	0.75	−3.06	3/1	1.95 2.06 1.95 1.96(C)
Co/2-C_3_N_4_-1	0.80	−3.84	4/2	2.08 1.96 2.05 2.02 1.95(C) 1.95(C)
Co/2-C_3_N_4_-2	0.77	−3.92	3/3	1.94 1.91 1.98 2.10(C) 2.06(C) 2.48(C)
Co/2-C_3_N_4_-3	0.77	−3.87	3/2	1.96 1.95 1.90 1.96(C) 2.09(C)
Co/2-C_3_N_4_-4	0.82	−3.66	3/1	2.04 1.92 1.97 1.97(C)

The binding energies of Co interacting with two melon layers ranged from −3.61 to −3.35 eV. Co is therefore more stable interacting with two melon layers compared to just one melon layer, although the binding energies were not drastically more exothermic (−3.61 eV for for Co/2-melon-2 and -3.00 eV for Co/melon-1). Co binding with two-layer melon formed either three or four Co–N bonds with both the layers, and up to two Co–C bonds. The most stable configuration was Co/2-melon-2 (*E*_binding_ = −3.61 eV), where the Co was coordinated to three N atoms and one C atom, having a square planar structure. The local geometries are listed in Table S4† and shown in Fig. S4,† indicating a variety of Co complexes, including linear, trigonal planar, square pyramidal, square planar, and tetrahedral. We also observed strained square pyramidal (Co/2-melon-1 and Co/2-melon-3, both with four Co–N and one Co–C bonds), and tetrahedral (Co/2-melon-4 with four Co–N bonds) geometries. Our calculations show that Co binding to two-layer melon tended to form Co–C bonds, whereas Co binding to single layer melon did not form any Co–C bonds.

#### 3.1.3 Binding of Co to fully condensed polymeric carbon nitrides

**Fig. 4:**
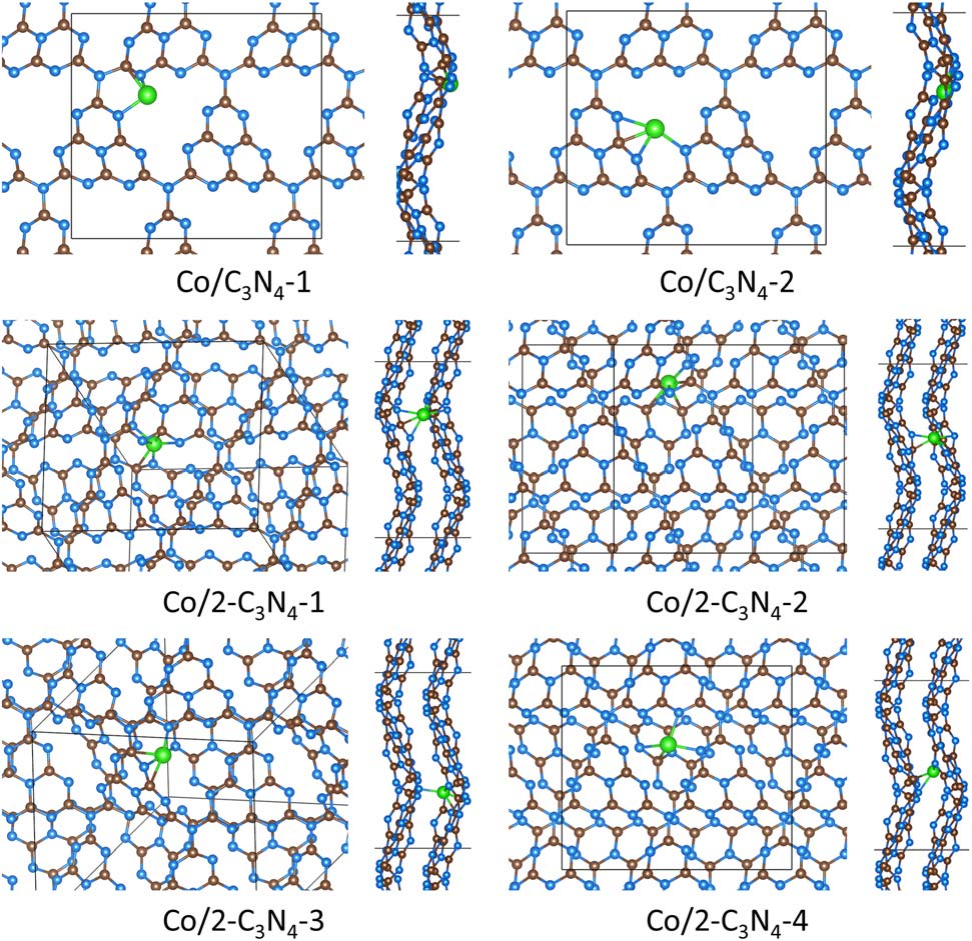
Stable structures where Co was bound to g-C_3_N_4_. Brown spheres represent carbon atoms, blue spheres represent nitrogen atoms, pink spheres represent hydrogen atoms, and green spheres represent cobalt atoms. When two g-C_3_N_4_ sheets are present, these are designated by the prefix ’2-’. Each unique configuration is identified by the final number (*e.g.*, ’-1′, ’-2′, *etc.*). Top and side views are shown. Simplified images of Co/C_3_N_4_ structures are found in Fig. S4.†

Following the modeling of Co/melon structures, we explored Co binding with fully polymerized carbon nitride g-C_3_N_4_. Fig. 4 shows results for Co bound to polymeric (periodic) g-C_3_N_4_, and details of these calculations are summarized in Table 2. We found two stable Co/C_3_N_4_ structures. Co/C_3_N_4_-1 had a binding energy of −2.88 eV. Co/C_3_N_4_-2 was the most stable g-C_3_N_4_ structure identified (*E*_binding_ = −3.06 eV). We also modeled Co binding with two-layer g-C_3_N_4_, and the binding energies were between −3.92 and −3.66 eV. The coordination numbers were between three and four for single-layer C_3_N_4_, and between four and six for two-layer C_3_N_4_. Notably, all the stable geometries with Co bound to C_3_N_4_ had Co–C bonds (one to three Co–C bonds). It is also worth mentioning that Co binding with 2 molecular carbon nitrides is about two times more stable than Co binding to just one molecule; however, Co binding with 2-layer C_3_N_4_ is only ∼ 30% more stable than Co binding with 1-layer C_3_N_4_. For example, there is not a drastic change in coordination for Co/C_3_N_4_-2 (four coordination with 3 Co–N bonds) compared to Co/2-C_3_N_4_-2 (six coordination with 3 Co–N bonds). Coordination geometries of all these structures are listed in Table S4, and shown in Fig. S4.†

#### 3.1.4 Connection to experimental work

Some experimental work has suggested four-coordinated bonding with N (Co–N_4_) when Co interacts with polymeric carbon nitrides.^61,107,109^ We observed four-coordinated Co in Co/C_3_N_4_-2, where 3 Co–N and 1 Co–C bonds formed. Fig. S1† shows that Co/2-melem-6 forms a Co–N_4_ arrangement, although this structure was less stable than other melem structures. In our previous work,^74^ we identified a similar possible Co–N_4_ geometry, although this involved Co binding with two heptazine molecules (chosen to mimic carbon nitride edges), rather than melem, melon, or g-C_3_N_4_. It should be noted that experimental work, such as Extended X-ray Absorption Fine Structure (EXAFS), may have trouble distinguishing between Co–N and Co–C bonds.^110–113^ Thus experimental work from literature may be observing both Co–N and Co–C bonds (such as in Co/C_3_N_4_-2, or Co/2-melon-2) or Co interacting with carbon nitride edges (such as in our previous work^74^). There may also be fitting errors in methods such as EXAFS due to low metal concentration.^113^ There is some literature that suggests the formation of M–C bonds. Fao *et al.*'s computation work^114^ reported Co coordinated to three N atoms and one C atom with a single-layer corrugated g-C_3_N_4_. Zheng *et al.*'s computation work^75^ found Co binding with corrugated g-C_3_N_4_ to form three Co–N bonds and two Co–C bonds, although no bond lengths were reported. Wang *et al.*'s work^115^ combined experimental results, EXAFS and X-ray photoelectron spectroscopy (XPS), with DFT results to show Cu binding to g-C_3_N_4_ forming one Cu–C and two Cu–N bonds.

Identification of the exact carbon nitride structure from experiment is not trivial, and it is possible that some literature work has misidentified their carbon nitride structure. See the introduction for discussion on partially condensed carbon nitrides and the difficulty of synthesizing g-C_3_N_4_. For example, temperature plays a vital role in what carbon nitride structure is synthesized and observed.^40^ It is possible that experimental papers finding four-coordinated Co atoms could be Co binding to partially condensed carbon nitrides. For example, Huang *et al.*'s experimental work^116^ argued that Co formed four identical Co–N bonds when incorporating Co into a carbon nitride. Their experimental work identified a significant number of NH_2_ groups in the carbon nitride, which suggests a structure other than fully condensed g-C_3_N_4_. This configuration resembles Co/2-melem-dimer-2 found in our study, as our structure also had four identical Co–N bonds. Ding *et al.*'s work^109^ presented a symmetric Co–N_4_ structure with 1.98 Å, similar to Co/2-melem-dimer-1, which has four Co–N bonds all similar in length. However, in Xiong *et al.*'s work,^117^ their experimental results indicted a Co coordination of 2.3, with lengths of 1.95 Å, similar to several of our modeled carbon nitrides: Co/melem-2, Co/2-melem-1, or Co/melem-dimer.

### 3.2 Electronic properties of Co-carbon nitride materials

#### 3.2.1 Bader charge analysis

We further examined the electronic properties of the Co/carbon nitride structures. Tables 1, 2, and S4† provide Co Bader charges of different structures, which indicate the oxidation state of the Co atoms. Co became positively charged in all cases once it interacted with the carbon nitride supports. This agrees with experimental^61,67,118^ and computational^75,119^ observations on the cationic nature of Co. Larger Co Bader charge indicated more oxidation of Co, and presumably more chemical interactions between the Co and carbon nitride substrate. The Co charges in Co/molecular carbon nitrides ranged from +0.1 to +0.96 |*e*^−^|, with an average value of +0.55 |*e*^−^|. With polymeric carbon nitrides, Co charges were similar for single-layer and two-layer structures, ranging from +0.72 to +0.86 |*e*^−^|, except for Co/melon-1, which had a Co charge of + 0.49 |*e*^−^|. The average Co charge with the polymeric carbon nitrides was +0.75 |*e*^−^|. Thus, the Co atoms were all cationic when bound to carbon nitride molecules, and Co atoms bound to polymeric carbon nitrides were generally more cationic than Co bound to molecular carbon nitrides.

**Fig. 5:**
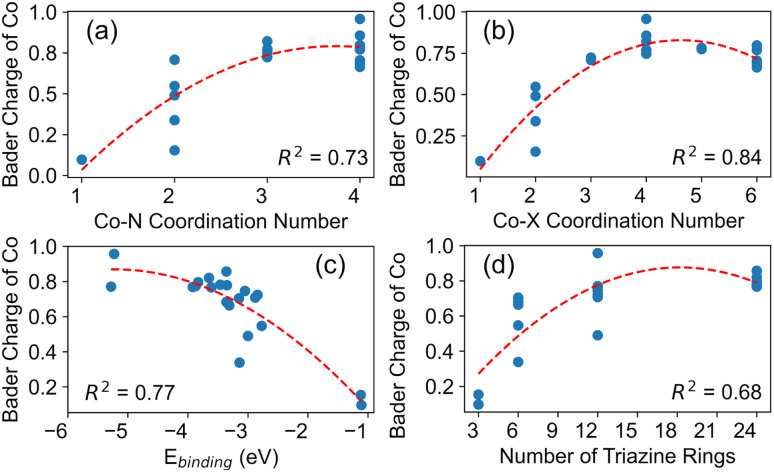
Correlations between the Bader charges of Co atoms and (a) Co–N coordination numbers, (b) total Co coordination numbers (both C and N), (c) Co binding energies, and (d) size of the carbon nitride (*i.e.*, number of triazine rings). The red dashed lines are second-order polynomial fits.

We next determined the relationships between the Co charges and structure of the complexes. Fig. 5 illustrates how the charges correlate to Co coordination number, binding energies, and structure of the carbon nitride support. The plots (a) and (b) show general trends that the Co charge increases (*i.e.*, becomes more oxidized) with both increasing Co–N coordination number and total Co coordination number. This connects with our observation that the Co charge was larger when Co was bound to more molecules (*e.g.*, two melem molecules compared to one molecule), indicating that more chemical interactions (as evidenced through increased coordination number) lead to stronger charge transfer between Co and neighboring atoms. Indeed, Fig. S2† demonstrates how coordination number correlates to binding energy. Plot (c) shows that the Co charge increases with stronger binding (*i.e.*, more negative binding energy). These results again indicate that stronger interactions between the Co atom and nearby bonding atoms lead to more charge transfer. Finally, we show in plot (d) how the size of the carbon nitride substrate correlates to Co charge. Larger carbon nitrides have more triazine rings, and generally bind stronger to Co (see Table 2), leading to more oxidation of the Co atom. Our results connecting the Co charge to structure are especially important in deciphering experimental data from SACs supported on carbon nitrides. For example, these trends could help interpret coordination numbers and/or oxidation states obtained through methods, such as from XAS.^120^ Indeed, the oxidation state of Co has been linked to its reactivity.^67^

#### 3.2.2 Band gaps

A key property for photocatalysis is the band gap, as a lower band gaps enables a broader spectrum of light to initiate photoexcitation. Co atoms may create favorable reaction sites (as discussed in Section 3.3), but also facilitate increased photoexcitation in Co/carbon nitride photocatalysts. Experimental work shows Co supported on carbon nitrides leads to a reduction in band gaps.,^118,121–123^ although the exact structures of the carbon nitrides in several of these papers still need clarification. The reduction of the band gap caused by binding of a Co atom to carbon nitrides has also been demonstrated in computational work involving g-C_3_N_4_.^78,79^ On the other hand, computational work on the effect of Co paired with other carbon nitrides (such as melem or melon) is missing. Therefore, we calculated band gaps of Co interacting with a variety of carbon nitrides.

**Fig. 6:**
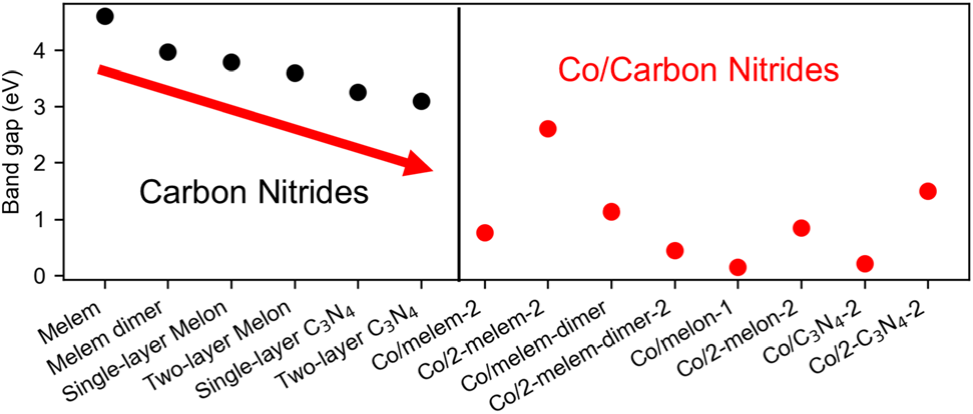
Band gaps of pristine carbon nitrides and the most stable Co/carbon nitrides calculated using the HSE06 functional. The left graph shows the band gaps for pristine carbon nitrides, while the right graph shows the band gaps for the most stable Co/carbon nitrides. The arrow shows the trends in band gaps for carbon nitrides, which decrease with increasing polymerization.

We show the band gaps of pristine carbon nitrides and the most stable Co/carbon nitrides calculated using the HSE06 hybrid functional in Fig. 6. Our results demonstrate that the band gap decreases with increased polymerization of the carbon nitrides (see left panel of Fig. 6), as already suggested in literature.^36^ Notably, the incorporation of a Co atom significantly reduces the band gaps (see right panel of Fig. 6). For example, the band gap of Co/2-melem-2 is 2.61 eV, which is 2 eV lower than a pristine melem molecule. Other Co/carbon nitride band gaps are much smaller, being in the range of 0.15 to 1.49 eV. There does not appear to be a clear relationship between carbon nitride size and Co/carbon nitride band gap. All Co/carbon nitrides have band gaps < 1.2 eV, with the exception of Co/2-melem-2 (2.61 eV) and Co/2-C_3_N_4_-2 (1.49 eV). However, our results do show that Co does indeed cause band gap reduction for Co supported on a variety of carbon nitrides.

#### 3.2.3 Electronic states

In the following section we briefly analyze the electronic states to better understand how Co leads to diminished band gaps and better photocatalysis. We show the density of states (DOS) using the HSE06 functional of carbon nitride molecules in Fig. S6 and polymers in S7.† The valence bands of the bare carbon nitrides consist mainly of states from nitrogen, and the conduction bands consist of states from carbon and nitrogen. Upon addition of Co, however, states arise in the carbon nitride band gaps consisting primarily of Co electronic states. These states effectively lower the band gaps as lower energies are needed to excite electrons from these gap states to the conduction bands. Previous literature indicated that similar gap states could arise over g-C_3_N_4_,^124^ and a melon-like structures.^82^ Our work shows that these gap states arise, regardless of the degree of polymerization (*i.e.*, molecular, partially condensed, and fully condensed carbon nitrides). Experiment also indicates that Co lowers electron–hole recombination when added to carbon nitrides.^123^ This lowered recombination could be facilitated by the midgap states created by Co in all carbon nitrides.

### 3.3 CO_2_ activation over Co–carbon nitride materials

CO_2_ photoreduction involves many different steps, and a key step is the activation of CO_2_. The formation of bent, anionic CO_2_ is important, as this enables the further reactivity of this otherwise stable linear molecule.^125^ If formation of bent, adsorbed CO_2_ is unfavorable, then CO_2_ reduction will be slow or hindered. We note that CO_2_ adsorption is weak on pure carbon nitrides, as the process has been reported for single-layer g-C_3_N_4_ to be slightly exothermic (−0.42 eV)^126^ or endothermic (0.02 eV,^78^ and 0.24 eV).^72^ Thus, improving the adsorption and reactivity of carbon nitrides is needed, such as pairing them with SACs. Experimental work on Co/carbon nitride catalysts shows these to be potential catalysts for CO_2_ reduction.^67,68,71–74^ In this section we examine the formation of activated CO_2_ over the various Co/carbon nitride structures.

#### 3.3.1 Formation of bent CO_2_

**Fig. 7:**
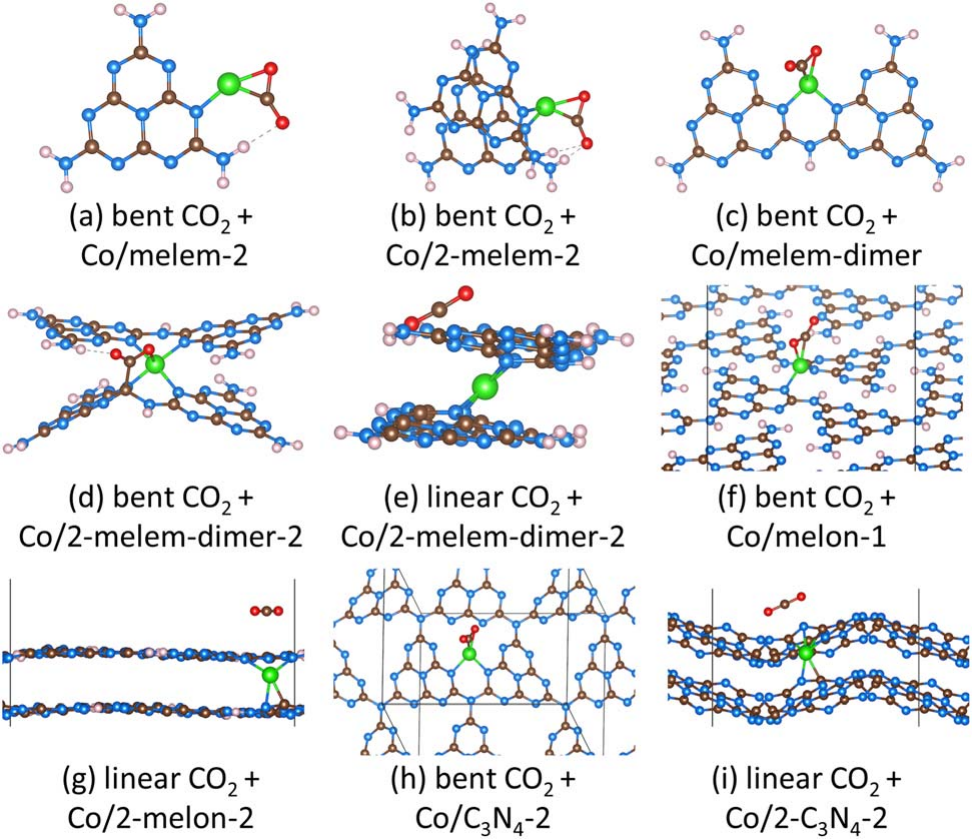
Most stable structures after CO_2_ adsorption to Co–carbon nitride materials. Shown are (a) bent CO_2_ + Co/melem-2, (b) bent CO_2_ + Co/2-melem-2, (c) bent CO_2_ + Co/melem-dimer, (d) bent CO_2_ + Co/2-melem-dimer-2, (e) linear CO_2_ + Co/2-melem-dimer-2, (f) bent CO_2_ + Co/melon-1, (g) linear CO_2_ + Co/2-melon-2, (h) bent CO_2_ + Co/C_3_N_4_-2, and (i) linear CO_2_ + Co/2-C_3_N_4_-2. Brown spheres represent carbon atoms, blue spheres represent nitrogen atoms, pink spheres represent hydrogen atoms, red spheres represent oxygen atoms, and green spheres represent cobalt atoms.

We modeled both linear and bent CO_2_ adsorbed on our most stable Co/CN structures. Fig. 7 presents only the most stable configurations (*i.e.*, lowest CO_2_ adsorption energies) for adsorbed CO_2_, while Fig. S10† shows all the CO_2_ structures we identified. Table 3 also provides adsorption energies and Bader charges for linear and bent CO_2_ on the different Co/CN structures. All the Co/carbon nitrides activated CO_2_, except for two-layer melon (Co/2-melon-2) and two-layer C_3_N_4_ (Co/2-C_3_N_4_-2). Several adsorption energies of bent CO_2_ were quite exothermic (from −2.33 to −1.12 eV), including Co/melem-2, Co/2-melem-2, and Co/melem-dimer. In all cases of bent CO_2_, the CO_2_ molecules exhibited O–C–O angles between 128.5° to 145.0°, and the C–O bonds were elongated to 1.20–1.30 Å (compared to the gas-phase CO_2_ bond length of 1.17 Å), indicating activated states, similar to previous work on other materials.^127–129^ The Bader charge analysis also shows that bent CO_2_ molecules were negatively charged from −0.74 |*e*^−^| to −0.47 |*e*^−^|, while the linear CO_2_ molecules were close to neutral (−0.10 |*e*^−^| to 0.00 |*e*^−^|). This again, confirms the anionic, activated state of bent CO_2_.

**Table 3:** Adsorption energies and deformation energies of linear and bent CO_2_ on different Co/carbon nitride systems. The most stable CO_2_ for each Co/carbon nitride is marked in gray. *E*_ads_ refers to the adsorption energy, *E*_Co/CN deform_ is the deformation energy of the Co/carbon nitride *E*_CO_2_deform_, represents the deformation energy of CO_2_, and *E*_int_ is the interaction energy between the adsorbent and CO_2_

Co/CN structures	CO_2_ configuration	O–C–O angle (°)	CO_2_ charge |*e*^−^|	*E*_ads_ (eV)	*E*_Co/CN deform_ (eV)	*E*_CO_2_deform_ (eV)	*E*_int_ (eV)
Co/melem-2	Bent	141.0	−0.67	−2.33	−0.03	1.55	−3.85
	Linear	177.7	−0.03	−0.16	0.01	0.01	−0.18
Co/2-melem-2	Bent	139.0	−0.74	−1.12	1.29	1.57	−3.98
	Linear	176.9	−0.02	−0.23	0.03	0.01	−0.27
Co/melem-dimer	Bent	141.6	−0.60	−1.29	0.22	1.38	−2.89
	Linear	177.7	−0.10	−0.44	0.03	0.01	−0.48
Co/2-melem-dimer-2	Bent	128.5	−0.63	−0.38	1.22	2.25	−3.86
	Linear	175.7	−0.04	−0.38	−0.01	0.01	−0.38
Co/melon-1	Bent	141.1	−0.60	−0.81	0.66	1.44	−2.91
	Linear	180.0	0.00	−0.05	0.00	0.00	−0.05
Co/2-melon-2	Bent	141.7	−0.60	0.02	0.74	1.21	−1.93
	Linear	179.1	0.00	−0.21	0.00	0.00	−0.20
Co/C_3_N_4_-2	Bent	145.0	−0.47	−0.79	0.34	1.17	−2.30
	Linear	175.8	−0.01	−0.31	0.11	0.02	−0.45
Co/2-C_3_N_4_-2	Bent	141.5	−0.57	1.13	2.00	1.28	−2.16
	Linear	179.0	0.00	−0.31	0.00	0.00	−0.31

These results demonstrated that Co could significantly improve CO_2_ adsorption and activation on carbon nitride materials. The Co atoms can facilitate charge transfer and serve as active centers to adsorb and activate CO_2_ molecules. As we mentioned previously, adsorption of CO_2_ on pristine g-C_3_N_4_,^72,78,126,130^ and melon^131^ was reported to form linear, weakly bound CO_2_. Co/molecular carbon nitrides and Co/single-layer polymeric carbon nitrides could however activate CO_2_ into bent, exothermic structures. The adsorption energies of linear and bent CO_2_ over Co/2-melem-dimer-2 were equivalent to each other. We modeled both linear and bent CO_2_ adsorbed to the Co atom in several different orientations. CO_2_, however, was only weakly absorbed to Co in the bent structure (Fig. 7d), likely due to steric hindrance as the CO_2_ situated between two carbon nitride molecules. In the linear structure, the Co atom preferred to interact only with the carbon nitride (Fig. 7e), despite the initial geometry with CO_2_ placed interacting with the Co atom. Again, steric hindrance prevents linear CO_2_ from interacting with the Co atom. In addition, we found that Co/two-layer polymeric carbon nitrides (melon and g-C_3_N_4_) only weakly absorbed linear CO_2_, and activation of CO_2_ into the bent form was endothermic. The Co atoms are sandwiched between the two carbon nitride layers for both carbon nitrides. A linear CO_2_ molecule placed between the two carbon nitride layers, to interact with the Co atom, was sterically hindered, and the CO_2_ molecules moved to the locations shown in Fig. 7g and i. Our efforts to form bent CO_2_ in these two-layered structures led to displacement of Co, and weakening of Co-carbon nitride interactions, as illustrated in Fig. S10.† As shown, in Co/2-melon-2 the Co atom migrates away from one melon layer to bond with a bent CO_2_. The Co atom after CO_2_ adsorption only interacts with one carbon nitride layer, and is therefore weakened compared to its pre-adsorbed state (Co interacting with two carbon nitride layers). In the case of Co/2-C_3_N_4_-2, an adsorbed, bent CO_2_ interacts with the Co atom as it sits between the two carbon nitride layers. The CO_2_ molecule is thus confined between the two layers and experiences steric repulsion, making activation of CO_2_ unfavorable.

**Fig. 8:**
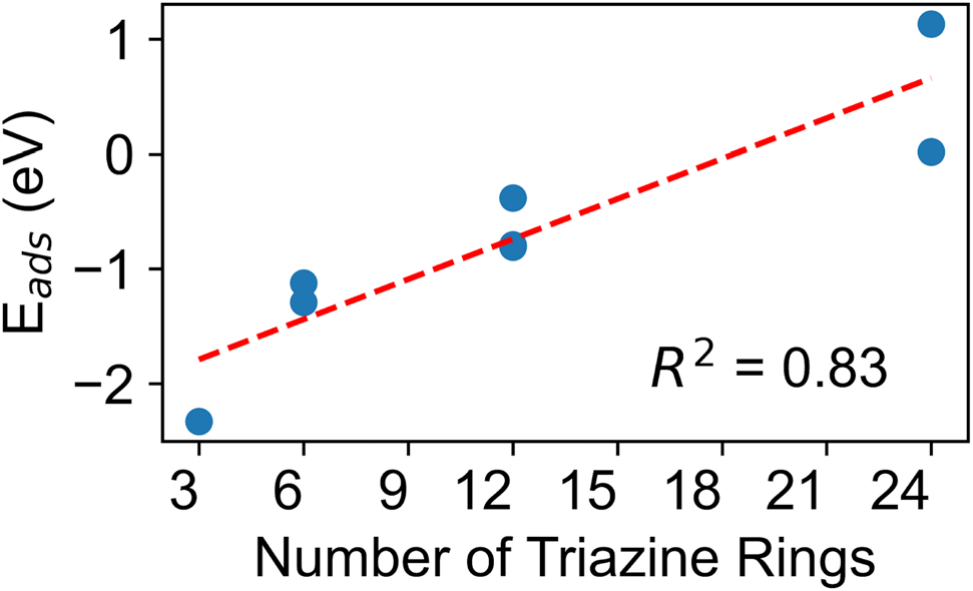
Correlation between the adsorption energies of CO_2_ over Co/carbon nitrides and the size of the carbon nitride (*i.e.*, number of triazine rings). The red dashed line shows a linear fit.

In order to further understand the role of the carbon nitride support, we plotted the stable bent CO_2_ adsorption energies *versus* carbon nitride size in Fig. 8. These results show that smaller carbon nitride supports have the strongest adsorption of bent CO_2_, while larger carbon nitrides have the weakest CO_2_ adsorption energies. The Sabatier principle prescribes that reactant adsorption energies should neither be too strong or too weak. In this case, intermediate adsorption energies occur for Co/melon-1 (−0.81 eV), Co/2-melem-2 (−1.12 eV), and Co/melem-dimer (−1.29 eV). Thus, based on the Sabatier principle, some molecular and partially condensed complexes may give the best CO_2_ reduction activity, although further clarification of the reaction mechanism is needed. We should also note that molecular carbon nitrides or melon strands may be representative of exposed carbon nitride edges, which may be a very reactive region.^132^

#### 3.3.2 The nature of linear and activated CO_2_

Generally bent, activated CO_2_ was more stable on the Co/carbon nitrides compared to linear CO_2_, with the exception of two-layer structures, Co/2-melon-2 and Co/2-C_3_N_4_-2. We next analyze the different structures to further explain the nature of linear *versus* bent CO_2_. For bent CO_2_ there were significant changes in Co geometry after CO_2_ adsorption. For structures that activated CO_2_ (all but two-layer carbon nitrides), we observed elongation of the original Co–N bonds and breaking of the Co–C bonds. But, there were also newly formed Co–C and Co–O bonds with the CO_2_ molecule. For example, in Co/2-melem-2, the Co atom had four Co–N and two Co–C bonds. After CO_2_ absorbed, the number of Co–N bonds reduced to two, and the two Co–C bonds broke, while new Co–C and Co–O bonds formed with CO_2_. For structures that favorably bound linear CO_2_ (Fig. 7g and i), the geometries and Co coordination environment remained essentially the same as the pre-adsorption state. We also found that Co became more cationic upon interacting with CO_2_. For instance, the average charge of Co over carbon nitrides that activated bent CO_2_ increased by +0.22|*e*^−^|. On the other hand, the average Co charge for those structures that preferred linear CO_2_ did not change, again indicating weak interactions.

In order to better understand why certain Co/carbon nitrides preferred linear over bent CO_2_, we examined the deformation and interaction energies of the complexes, which are listed in Table 3. The adsorption energy reflects a balance between the deformation of both the adsorbate and substrate upon adsorption, and their chemical interactions. The deformation energies embody the geometric changes of the adsorbate and substrate from their pre-adsorbed to post-adsorbed states. The interaction energy represents the bond formation energy between the adsorbate and substrate.^133–136^ The adsorption energy can be written as: Δ*E*_ads_ = *E*_int_ + *E*_Co/CNdeform_ + *E*_CO_2_deform_. Thus, whether adsorption is exothermic or not depends on the nature of structural changes upon adsorption and the nature of favorable (or unfavorable) electronic interactions. Deformation energies (*E*_deform_) of Co/carbon nitrides and CO_2_ adsorption were calculated by eqn (3) and (4):3
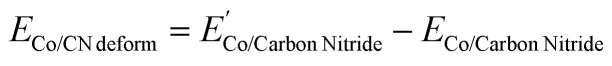
4
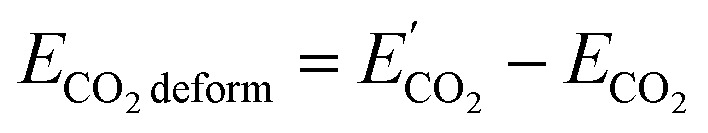
Here 
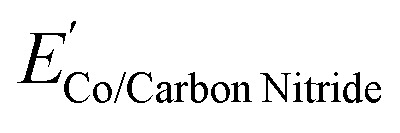
 is the energy of the Co/carbon nitride complex after CO_2_ adsorption, but with CO_2_ removed. *E*_Co/Carbon Nitride_ is the energy of the geometry of the optimized complex with no CO_2_ present. *E*′ is the energy of the adsorbed CO_2_ minus the Co/carbon nitride complex. *E*_CO_2__ is the energy of gas-phase CO_2_. The interaction energy (*E*_int_) was calculated by eqn (5):

5 *E*_int_ = Δ*E*_ads_ − *E*_Co/CN deform_ − *E*_CO_2_deform_

CO_2_ deformation energies were always positive for bent adsorption (average value of 1.48 eV), indicating an energy cost to bend the molecule into its activated state. The Co/carbon nitride deformation energies for the bent CO_2_ case were quite varied, ranging from −0.03 to 2.00 eV, with an average value of 0.81 eV. Since deformation energies for the bent CO_2_ case were positive, interaction energies must be sufficiently negative for adsorption to be exothermic. Indeed, our results show this to be the case, as these interactions energies were all negative (ranging from −1.93 to −3.98 eV). This strong interaction between bent CO_2_ and the Co/carbon nitride overcomes geometric distortions to enable exothermic adsorption of CO_2_. Indeed, bent CO_2_ becomes anionic, further indicating strong interactions occurring with bent CO_2_. The significantly more exothermic adsorption energies of bent CO_2_ molecules on Co/melem-2, Co/2-melem-2, and Co/melem-dimer can be attributed to their more negative interaction energies (from −2.89 to −3.98 eV) compared to Co/melon-1 and Co/C_3_N_4_-2 (−2.91 eV and −2.30 eV).

On the other hand, interaction energies for linear CO_2_ were much weaker, ranging from −0.05 to −0.48 eV. Despite CO_2_ deformation energies for linear CO_2_ being close to zero, the interaction energies were not exothermic enough to lead to strong linear CO_2_ adsorption. CO_2_ charges were close to neutral for linear CO_2_, indicating little charge transfer and weak interactions between CO_2_ and the Co/carbon nitrides.

**Fig. 9:**
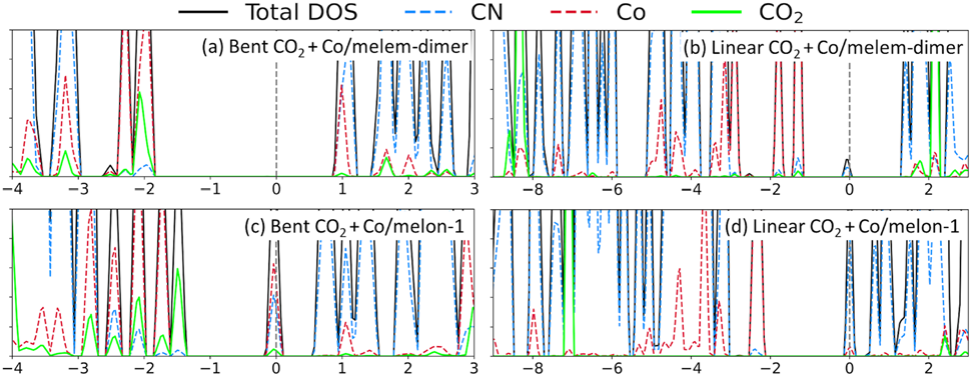
Example density of states (DOS) plots for adsprbed CO_2_, indicating how linear and bent CO_2_ interact with Co/carbon nitrides: (a) bent CO_2_ + Co/melem-dimer, (b) linear CO_2_ + Co/melem-dimer, (c) bent CO_2_ + Co/melon-1, and (d) linear CO_2_ + Co/melon-1. The total, carbon nitride (CN), Co, and CO_2_ DOS are shown. The energies are relative to the Fermi level, which has been shifted to 0 eV and indicated by a dashed vertical line. Due to smearing of the electronic states, the Fermi level appears within the valence band, even though the HOMO energy level is below the Fermi level.

DOS plots also provide insight on the nature of interactions between CO_2_ and the Co/carbon nitride catalysts. We show in Fig. 9 example DOS plots for linear and bent CO_2_ interacting with different Co/carbon nitride complexes. Other DOS plots are shown in Fig. S8 and S9.† These plots show that CO_2_ in the bent position interacts strongly with Co. See for example the many overlapping peaks involving Co and CO_2_ near −2, −3.3, an −3.8 eV for Co/melem-dimer. Similar overlapping peaks occur at −1.8, −2.1, −2.5, and −2.9 eV for Co/melon-1. These interactions are due to bond formation between the CO_2_ molecule and the cobalt atom. On the other hand, such interactions are missing for linear CO_2_. The CO_2_ electronic levels are all much lower in energy and overlap with Co is minimized. Both the interactions energies and density of state plots confirm that bent CO_2_ forms strong bonds with the Co/carbon nitride while linear CO_2_ does not.

### 3.4 Implications for photocatalysis

Experimental work shows that addition of Co to carbon nitrides improves photoactivity.^67,78,116^ The exact structure of carbon nitride in these experiments is not fully resolved. We modeled non-polymerized, partially condensed, and fully condensed carbon nitrides, which should provide details on several possible carbon nitrides that could be present in real-world catalysts. Our results showed that Co/carbon nitrides have better photoexcitation compared to pristine carbon nitrides, through more narrow band gaps. This should lead to more photoexcited electrons that may enable photocatalytic reduction. Experiment also indicates that Co lowers electron–hole recombination when added to carbon nitrides.^123^ This lowered recombination could be facilitated by the mid-gap states created by Co, which we observed. Our results also indicate that in addition to facilitating a larger number of charge carriers to participate in photocatalytic reactions, Co/carbon nitride catalysts also are effective for CO_2_ activation. In particular, we found that molecular and partially condensed carbon nitride complexes with CO_2_ may have moderate CO_2_ adsorption energy (neither too strong nor too weak) to enable CO_2_ reduction. On the other hand, multi-layer carbon nitrides were found to have difficulties activating CO_2_. Future work may assess the full catalytic mechanism, but in the least, CO_2_ activation is a crucial reaction step which Co/carbon nitrides readily enable. All these different effects caused by Co paired with different carbon nitride supports (increased photoexcitation, decreased charge recombination, and increased reactivity) lead to better CO_2_ reduction and photocatalytic activity (as observed in experiments^67–74^).

## 4 Conclusions

Literature has largely focused on two-dimensional g-C_3_N_4_ as a support for SACs, despite evidence showing that partial condensation is likely common in working carbon nitride catalysts. We assessed single Co atoms supported on a variety of carbon nitrides as potential photocatalysts. We modeled single Co atoms on six different carbon nitrides with varying degrees of polymerization, including molecular (melem and melem dimer), partially condensed single-layer and two-layer melon, and fully condensed (single-layer and two-layer C_3_N_4_). Several stable Co/carbon nitrides were identified with Co forming up to four Co–N bonds, and three Co–C bonds having various Co local geometries. We also discovered correlations between Co coordination number, Co charge, and binding energy. As the Co coordination number increased, the Co atom generally became more oxidized, and the binding energy became more exothermic. Furthermore, binding of Co tended to be strongest with larger carbon nitrides.

In addition, we predicted how Co supported on a carbon nitride could function as a photocatalyst for CO_2_ reduction. Adding Co to the various carbon nitrides lowered the band gaps, which should lead to better photoexcitation yield. Co atoms also helped activate CO_2_ into a bent, anionic state, an essential step for CO_2_ reactivity. The activation of CO_2_ is facilitated by strong interactions between the CO_2_ molecule and Co/carbon nitride, which is required to overcome unfavorable bending of the CO_2_ molecule. For example, the interaction energy between a bent CO_2_ molecule and Co/melem-2 is −3.85 eV, while the deformation energy of the bent CO_2_ molecule is 1.55 eV, leading to an overall favorable activation of CO_2_. Exothermic bent CO_2_ adsorption energies varied between −2.33 and −0.38 eV, and likely a moderate CO_2_ adsorption energy (not too strong and not too weak, such as near −1 eV) would best facilitate CO_2_ reduction. Two melem molecules, a melem dimer, melon, and single-layer g-C_3_N_4_ all have moderate CO_2_ adsorption energies, and thus may be good candidates for CO_2_ photoreduction. Our work shows that carbon nitrides other than g-C_3_N_4_ when paired with single metal atoms have potential as photocatalysts, and therefore should be studied further.

## Conflicts of interest

There are no conflicts to declare.

## Supplementary Material

RA-015-D5RA03826J-s001

## Data Availability

Data from the various tables and figures for this article have been included in the ESI.† Simulation files, including geometries, can be found at https://github.com/Deskins-group/Structure-Files/tree/master/Co-Carbon-Nitrides.
