# Supplementary material for: Determining the structure and properties of CO2 reduction photocatalysts: single atom cobalt atoms supported on various carbon nitrides
Source: RSC Adv. 2025 Jul 7;15(29):23448–60. doi: 10.1039/d5ra03826j (PMC12230944; doi:10.1039/d5ra03826j)
Supplement: RA-015-D5RA03826J-s001 [file RA-015-D5RA03826J-s001.pdf]

Supplementary Information (SI) for RSC Advances.
This journal is © The Royal Society of Chemistry 2025

Electronic Supplementary Information (ESI) for RSC Advances

Determining the Structure and Properties of CO_2 Reduction Photocatalysts: Single Atom Cobalt Atoms Supported on Various Carbon Nitrides

Qian Qian
and
N. Aaron Deskins
Department of Chemical Engineering, Worcester Polytechnic Institute

1 Further Simulation Details

Various models of melon have been proposed in the literature, including the Fina, Lotsch, and S2 models[1]. Our results indicated that the Fina, Lotsch and S2 conformers were relatively close in energy (within 0.01 eV), similar to what Melissen's work suggested about these melon structures[1]. Therefore, we used Fina's melon model for simplicity and consistency. Our model of a single-layer melon sheet was based on the work of Fina et al.[1,2] They modeled a two-layer melon structure, which we took and removed one layer to obtain a single-layer sheet. Melon is more stable with a planar structure[3,4], and so we modeled all melon structures as flat (non-corrugated).

When optimizing the geometry of Co bound to the carbon nitride structures, we considered several different initial geometries. For example, when modeling Co/melem, we began our modeling by attaching a Co atom to a single molecular carbon nitride (either one melem or one melem dimer). Next, we modeled a Co atom bonded between two molecular carbon nitrides that were aligned in the same plane. To reduce repulsion among the NH_2 groups, we also rotated the molecular carbon nitrides. Additionally, we stacked molecular carbon nitrides to simulate Co binding to two-layer carbon nitrides. When modeling Co with melon, we began our modeling by positioning a Co atom at several locations along the space between two melon strands. Most literature modeled transition metals binding to a single-layer melon-like polymer within these empty spaces.[5,6] We modeled Co binding to two-layer melon structure at various positions between the two melon layers. We also shifted one layer of the two-layer melon in various translational directions to simulate layers being displaced during Co binding. When modeling Co with g-C_3N_4, we placed a Co atom in different locations in the pocket of the single-layer g-C_3N_4 structure. We also modeled a Co atom binding to two-layer g-C_3N_4 at different locations. For some of these calculations we implemented translational displacement between the two layers to simulate their shifting when binding with a Co atom.

We modeled CO_2 adsorbed to the Co/carbon nitride structures. We considered several possible initial geometries in order to determine the most stable adsorbed CO_2 configurations. This included linear CO_2 that approached the Co atom by either the C or O atom of CO_2. We also modeled bent CO_2, where a bent CO_2 approached the Co atom by either the C atom or both C/O atoms of CO_2.

We show in Table S1 simulations with different k-point meshes for periodic carbon nitride materials. The vacuum spacing was aligned in either the y- or z-direction, depending on the simulation. Because of the cell symmetry, changing the vacuum from the y- or z-direction involves

simply a rotation of the cell and the carbon nitride polymers. Thus, a 2x2x1 mesh (vacuum along z-direction) and 2x1x2 (vacuum along y-direction) are equivalent. The results show that using a 2x2x1 (or equivalently 2x1x2) k-point mesh, where the vacuum space has 1 k-point mesh, gave sufficiently converged energies. We therefore used 2x2x1 k-point meshes (or equivalently 2x1x2) for our simulations of periodic carbon nitrides.

Table S1: A comparison of calculated energies using different k-point meshes. Our results show that using a 2x2x1 k-point mesh (or equivalently 2x1x2 k-point mesh) gave the same energies as using 3x3x1 (or equivalently 3x1x3) k-point meshes.

Models	k-points	ΔE (eV)
Single-layer Melon	1x1x1	-567.05
Single-layer Melon	2x1x2	-567.10
Single-layer Melon	3x1x3	-567.10
Two-layer Melon	1x1x1	-1136.79
Two-layer Melon	2x1x2	-1136.89
Two-layer Melon	3x1x3	-1136.89
Single-layer g-C_3N_4	1x1x1	-479.34
Single-layer g-C_3N_4	2x2x1	-479.39
Single-layer g-C_3N_4	3x3x1	-479.39
Two-layer g-C_3N_4	1x1x1	-961.45
Two-layer g-C_3N_4	2x1x2	-961.55
Two-layer g-C_3N_4	3x1x3	-961.55

2 Comparison of Our Results with Literature

We present selected results comparing our data with literature data.

2.1 Bare Carbon Nitrides

We first modeled different possible carbon nitride models. Literature [1,4,7,8] reports that different carbon nitride structures may form, depending on synthesis procedures and conditions. Figure 1 in the main text shows all the different carbon nitride structures we modeled in this work. We compared the structural (bond length, bond angle, and lattice parameters if applicable) and electronic (e.g., band-gap) properties of optimized pristine carbon nitrides to literature and found agreement between our results and literature [1,4,7,9–11]. A detailed comparison is provided in Tables S2 (carbon nitride geometries) and S3 (carbon nitride band gaps). These results indicate the reliability of our modeling approach.

Table S2: A comparison of our calculated geometries with literature results. Lattice parameters (unit cell dimensions a, b, and c and angles α, β, and γ) and bond distances/angles (C-N_{3C} bond lengths and C-N_{3C}-C angles) are given. For the melon and g-C_3N_4 we included a vacuum space between the layers, while literature did not. Hence, one direction was always larger in our periodic calculations compared to literature.

Structure	Source	Lattice Parameters						Geometry Comparison	
		a	b	c	α	β	γ	C-N_{3C} Bond Length (Å)	C-N_{3C}-C Angle
Melem	Current Work	30.0	30.0	30.0	90.0	90.0	90.0	1.41, 1.41, 1.41	121, 121, 121
	Botari et al., 2017 [7]	-	-	-	-	-	-	1.41, 1.41, 1.41	121, 121, 121
Melem Dimer	Current Work	30.0	30.0	30.0	90.0	90.0	90.0	1.41, 1.41, 1.41	121, 121, 121
	Botari et al., 2017 [7]	-	-	-	-	-	-	1.41, 1.41, 1.41	121, 121, 121
Single-layer Melon	Current Work	12.8	29.8	16.8	90.0	90.0	90.0	1.40, 1.40, 1.42	120, 120, 122
	Melissen et al., 2016 [4]	12.7	16.8	15.0	90.0	90.0	90.0	1.39, 1.40, 1.41	120, 121, 122
Two-layer Melon	Current Work	12.7	29.9	16.7	90.1	90.0	90.0	1.40, 1.40, 1.42	120, 120, 122
	Melissen et al., 2021 [1]	12.7	6.4	16.7	90.0	90.0	90.0	1.39, 1.40, 1.41	120, 120, 122
Single-layer g-C_3N_4	Current Work	11.8	13.1	29.8	90.0	90.0	90.0	1.41, 1.41, 1.39	123, 123, 122
	Melissen et al., 2016 [4]	11.8	13.1	18.3	90.0	90.0	90.0	1.43, 1.62, 1.50	123, 166, 124
Two-layer g-C_3N_4	Current Work	11.9	30.0	13.3	90.0	90.0	89.8	1.40, 1.40, 1.39	123, 122, 123
	Botari et al., 2017 [7]	11.9	7.0	13.3	90.0	90.0	90.0	1.38, 1.39, 1.40	122, 122, 122

2.2 Co/Molecular Carbon Nitrides

We are not aware of any literature modeling Co interactions with a melem molecule.

2.3 Co/Partially Condensed Carbon Nitrides

Zheng et al's work [12] showed Co binding energies of -2.85 eV and -2.78 eV at the same locations as Co in our Co/melon-1 and Co/melon-2, which are very close to our values. Zheng et al's work also found Co binding to single-layer melon had a charge of +0.51 $|e^-|$, which is very close to our value of +0.49 $|e^-|$.

Table S3: Band gaps of various carbon nitride structures calculated using the PBE and HSE06 functionals, compared with literature values.

Structure	Source	Method	Band Gap (eV)
Melem	Current Work	PBE	3.48
	Lau et al., 2015[9]	PBE	3.50
Melem dimer	Current Work	PBE	2.92
	Lau et al., 2015[9]	PBE	2.95
Single-layer Melon	Current Work	HSE06	3.79
	Meek et al., 2014[10]	HSE06	3.64
Single-layer g-C_3N_4	Current Work	HSE06	3.26
	Wu et al., 2014[11]	HSE06	3.05

2.4 Co/Fully Condensed Carbon Nitrides

Fu et al's work[13] reported Co binding to the corner of a corrugated single-layer g-C_3N_4, which also resulted in two Co-N coordination, similar to our Co/C_3N_4-1 structure. Homlamai et al's work[14] reported a binding energy of -3.5 eV for Co binding to corrugated single-layer g-C_3N_4, relatively similar to our own value of -3.06 eV. Ghosh et al's work[15] however reported a binding energy of -1.4 eV for Co binding to single-layer g-C_3N_4. This difference is attributed to the GGA+U method used by Ghosh et al. For comparison with literature we also modeled Co binding to flat (planar) g-C_3N_4, and obtained a binding energy of -3.65 eV. Chen et al.[16] modeled Co binding to flat g-C_3N_4 and found a binding energy of -3.75 eV, similar to our value. Chen et al. used a modeling approach similar to our own (GGA functional, Grimme van der Waals corrections), and hence the similar binding energies.

We also compared our geometries with literature. Ghosh et al's work[15] reported six-coordinated Co when bound to corrugated single-layer g-C_3N_4, but several of their bonds were large and beyond the cutoff distance we used (2.39, 2.10, 2.10, 2.54, 2.77, and 2.65 Å). Their structure was also not as corrugated as our C_3N_4 sheet, which could have influenced their results. Gao et al.[17] modeled Pt bound to g-C_3N_4, and found that corrugation can have a significant effect on binding. Ao et al's work[18] presented Co binding to a corrugated single-layer g-C_3N_4 sheet with three Co-N bonds that were 1.96, 1.97, and 2.19 Å, which are very close to the Co-N bond lengths in Co/C_3N_4-2.

Our Co binding energies with two-layer g-C_3N_4 were between -3.66 and -3.92 eV. For comparison, An et al's work[19], modeling a single Fe atom binding between two layers of g-C_3N_4, found a binding energy of -3.92 eV, which is very close to our Co/2-C_3N_4-2 structure. Shen et el's work combined experimental and computational work and presented a Cu-N_3C_1 structure with a single Cu atom binding to two-layer g-C_3N_4.[20] We found similar Co coordination with two-layer g-C_3N_4, being 3/1, 3/2, 3/3, and 4/2 (first number Co-N coordination, second number Co-C coordination).

Ao et al's study[18] predicted a Co charge of + 0.82 $|e^-|$ when binding to corrugated single-layer g-C_3N_4, and we predicted a Co charge of + 0.75 $|e^-|$. Chen et al's work[16] found Co binding to single-layer planar g-C_3N_4 to have a charge of +0.84 $|e^-|$. Our simulation of Co binding to single-layer planar g-C_3N_4 gave a Co charge of + 0.76 $|e^-|$. Thus, we find similar Co charges to those reported literature values.

2.5 CO_2 Adsorption

Our bent CO_2 adsorption energy on Co/C_3N_4-2 was -0.79 eV, which is similar to other literature values over Co/C_3N_4. Previous modeling work showed that Co modified g-C_3N_4 could adsorb CO_2

and form a bent structure with an adsorption energy of -0.83 eV, and an O-C-O angle of 145.6°.[18] Similarly, Guo et al.'s work[21] reported bent CO_2 with an adsorption energy of -0.81 eV. Goliaei et al.'s work[22] reported a CO_2 adsorption energy of -0.84 eV on Co/g-C_3N_4. Fu et al.'s work[13] also reported bent CO_2 adsorbed on Co modified g-C_3N_4 with an adsorption energy of -0.07 eV, which is an outlier compared to previous work. Our linear adsorption energy was found to be -0.01 eV over Co/C_3N_4-2. This also agrees with literature. There is work[14] indicating linear CO_2 only weakly absorbed on Co/g-C_3N_4 with an adsorption energy of -0.16 eV. Tang et al's work[23] also reported weak adsorption of linear CO_2 on Co/g-C_3N_4 (adsorption energy of -0.03 eV). Adsorption of CO_2 over single-layer melon has also been reported to be slightly exothermic (-0.31 eV)[24].

3 Other Stable Co/Carbon Nitride Structures

Besides those structures discussed in the main text, we also identified other (typically less stable) Co/carbon nitride complexes. Figure S1 shows several such structures.

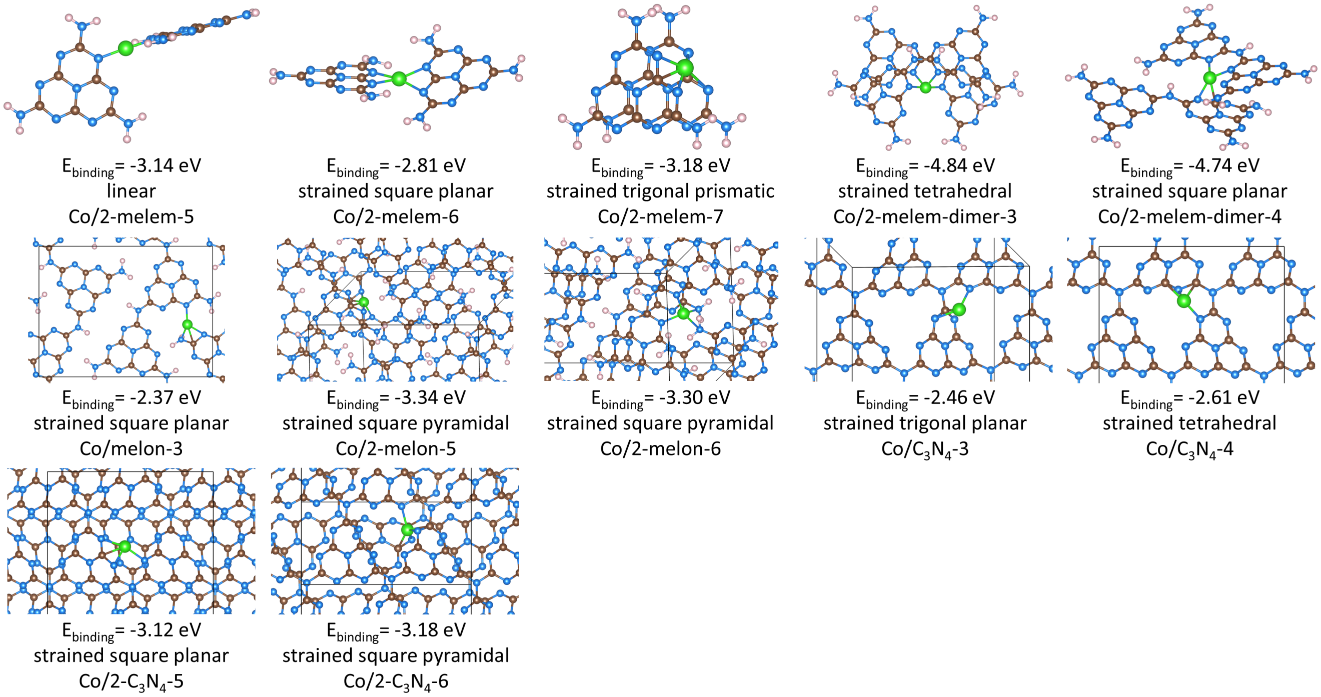

Figure S1: Other Co/carbon nitride structures identified in our modeling. Such structures are either less stable than those presented in the main text, or similar to those geometries. Brown spheres represent carbon atoms, blue spheres represent nitrogen atoms, green spheres represent cobalt atoms, and pink spheres represent hydrogen atoms.

4 Cobalt Coordination Number and Binding Energy

Figure S2 presents relationships between Co-nitrogen coordination number and binding energies, as well as total Co coordination number and binding energies.

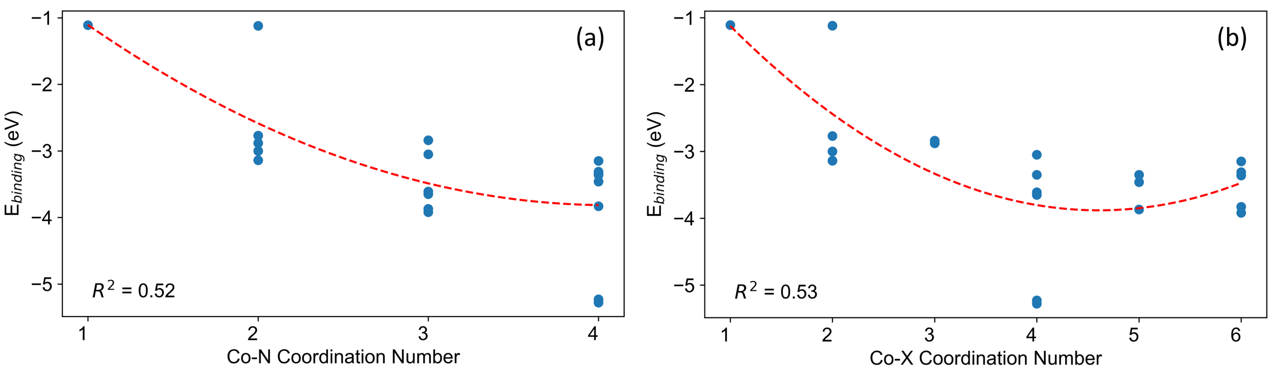

Figure S2: Illustration of how binding energies correlate to Co coordination numbers for structures identified and presented in the main text. (a) shows coordination to N only, while (b) shows coordination to C and N. The red dashed lines are second-order polynomial fits.

5 Co/Carbon Nitride Structures

Table S4 provides a summary of coordination numbers, Bader charges, and local geometries of Co interacting with different carbon nitrides. We show simplified diagrams of the various Co/carbon nitride structures in Figures S3 and S4, indicating the local geometry around the Co atoms.

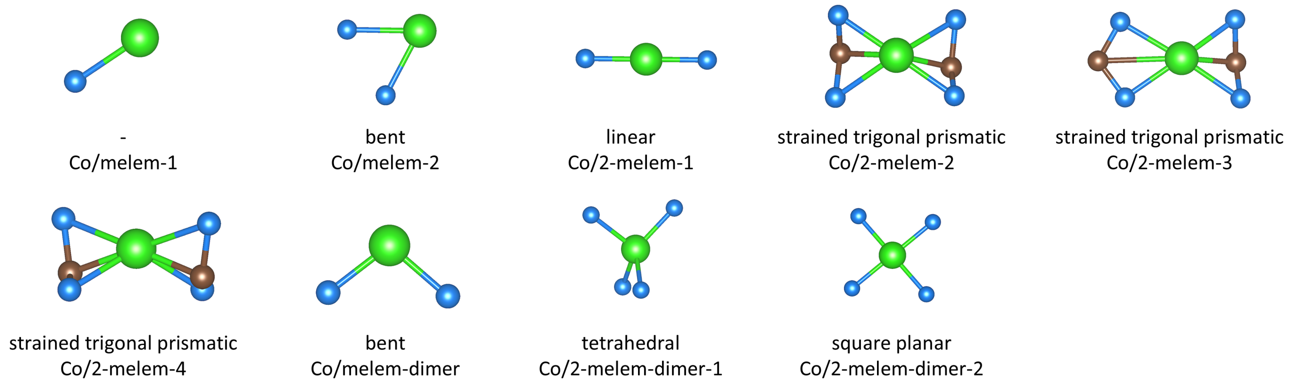

Figure S3: Simplified representations of the Co/molecular carbon nitrides indicating the structures and coordination of the Co atoms. Brown spheres represent carbon atoms, blue spheres represent nitrogen atoms, and green spheres represent cobalt atoms.

Table S4: A summary of Co charge, Co-N coordination number (CN), Co-C coordination number(CN), total coordination number(CN), and local geometry for each structure.

Models	Co Charge	Co-N CN	Co-C CN	Total CN	Co Coordination Geometry
Co/melem-1	0.10	1	0	1	-
Co/melem-2	0.15	2	0	2	bent
Co/2-melem-1	0.34	2	0	2	linear
Co/2-melem-2	0.68	4	2	6	strained trigonal prismatic
Co/2-melem-3	0.66	4	2	6	strained trigonal prismatic
Co/2-melem-4	0.70	4	2	6	strained trigonal prismatic
Co/melem-dimer-1	0.55	2	0	2	bent
Co/2-melem-dimer-1	0.96	4	0	4	tetrahedral
Co/2-melem-dimer-2	0.77	4	0	4	square planar
Co/melon-1	0.49	2	0	2	linear
Co/melon-2	0.72	3	0	3	trigonal planar
Co/2-melon-1	0.78	4	1	5	strained square pyramidal
Co/2-melon-2	0.77	3	1	4	square planar
Co/2-melon-3	0.78	4	1	5	strained square pyramidal
Co/2-melon-4	0.86	4	0	4	tetrahedral
Co/C_3N_4-1	0.71	2	1	3	strained trigonal planar
Co/C_3N_4-2	0.75	3	1	4	strained square planar
Co/2-C_3N_4-1	0.80	4	2	6	strained trigonal prismatic
Co/2-C_3N_4-2	0.77	3	3	6	strained trigonal prismatic
Co/2-C_3N_4-3	0.77	3	2	5	strained bipyramidal
Co/2-C_3N_4-4	0.82	3	1	4	strained square planar

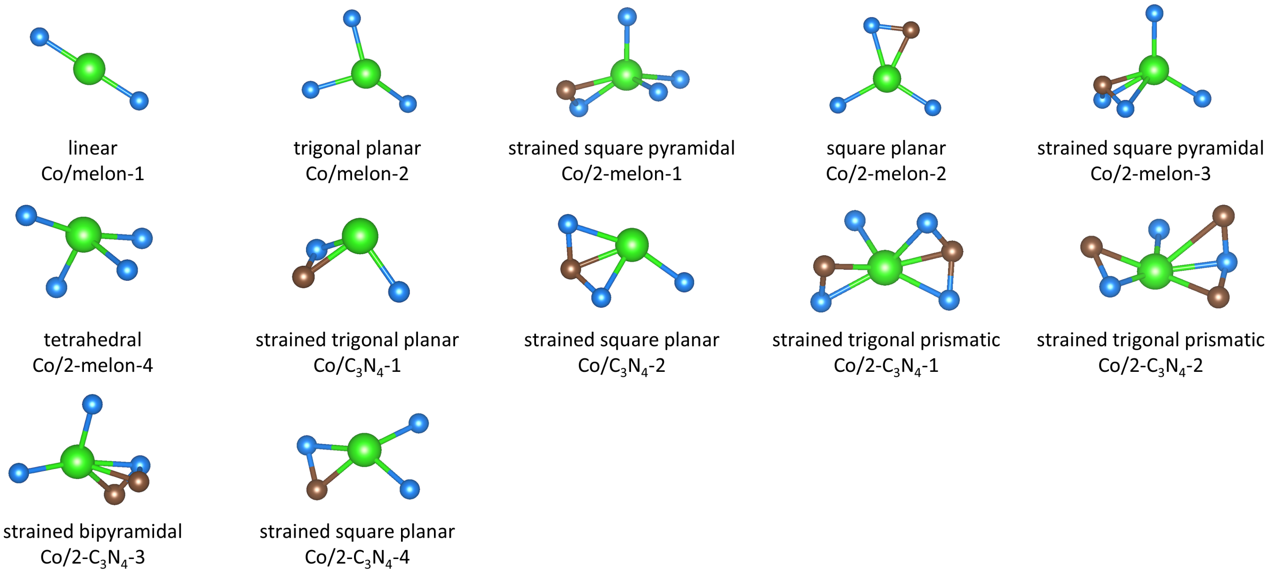

Figure S4: Simplified representations of the Co/polymeric carbon nitrides indicating the structures and coordination of the Co atoms. Brown spheres represent carbon atoms, blue spheres represent nitrogen atoms, and green spheres represent cobalt atoms.

6 Electronic Structure and Band Gaps

Our calculated band gaps of pristine carbon nitrides (shown in Figure 1) and all stable Co/carbon nitrides (shown in Figures 2-4) using HSE06 are provided in Figure S5. The band-gaps for melem, melem dimer, single-layer melon, and single-layer g-C_3N_4 are 4.61, 3.97, 3.79, and 3.26 eV, respectively. A comparison between band gaps of pristine carbon nitrides used in this work and literature values is provided in Table S3. Also shown our density of state plots for Co/carbon nitrides and adsorbed CO_2 structures (Figures S6 to S9).

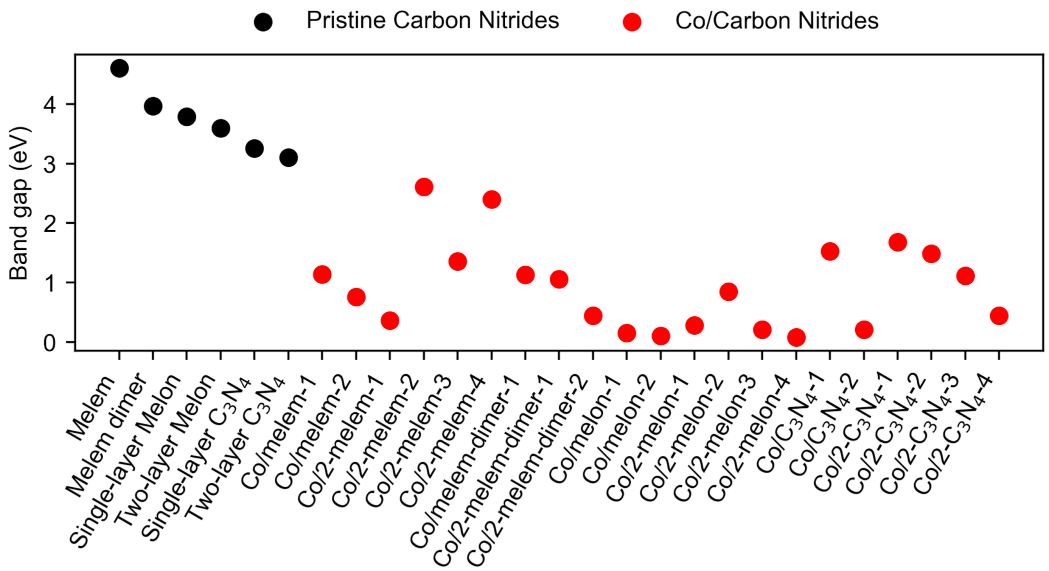

Figure S5: Calculated band gaps of various carbon nitrides and all stable Co/carbon nitride materials using the HSE06 functional. Black dots are for pristine carbon nitrides and red are for Co/ carbon nitrides.

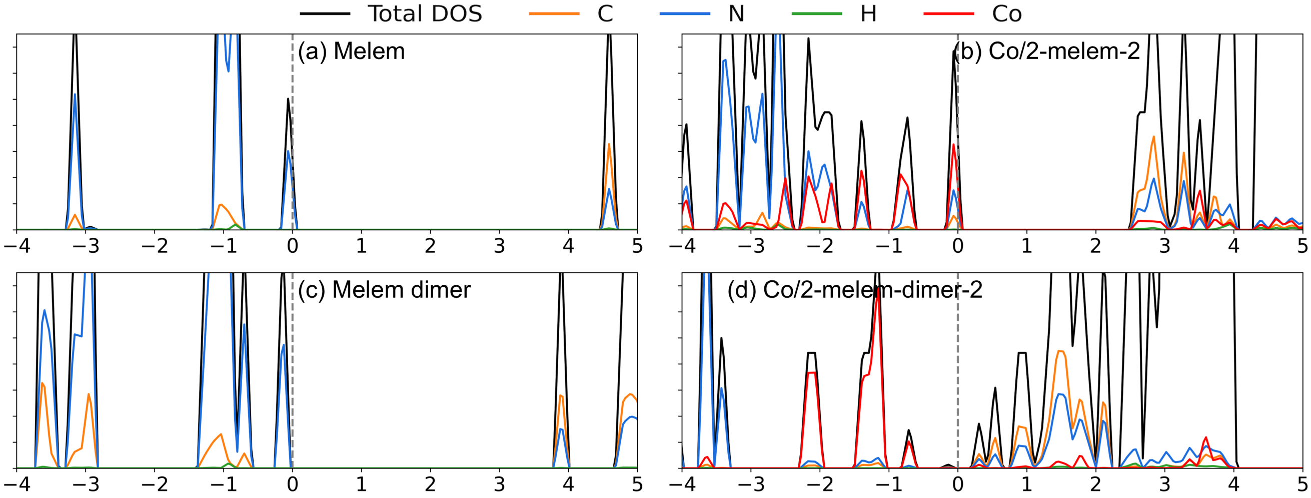

Figure S6: Density of states (DOS) plots for (a) melem, (c) melem dimer, and select Co-bound molecular carbon nitride structures, (b) and (d). These Co/melem complexes are the most stable of those we modeled. The energies are relative to the Fermi level, which has been shifted to 0 eV and indicated by a dashed vertical line. Due to smearing of the electronic states, the Fermi level appears within the valence band, even though the HOMO energy level is below the Fermi level.

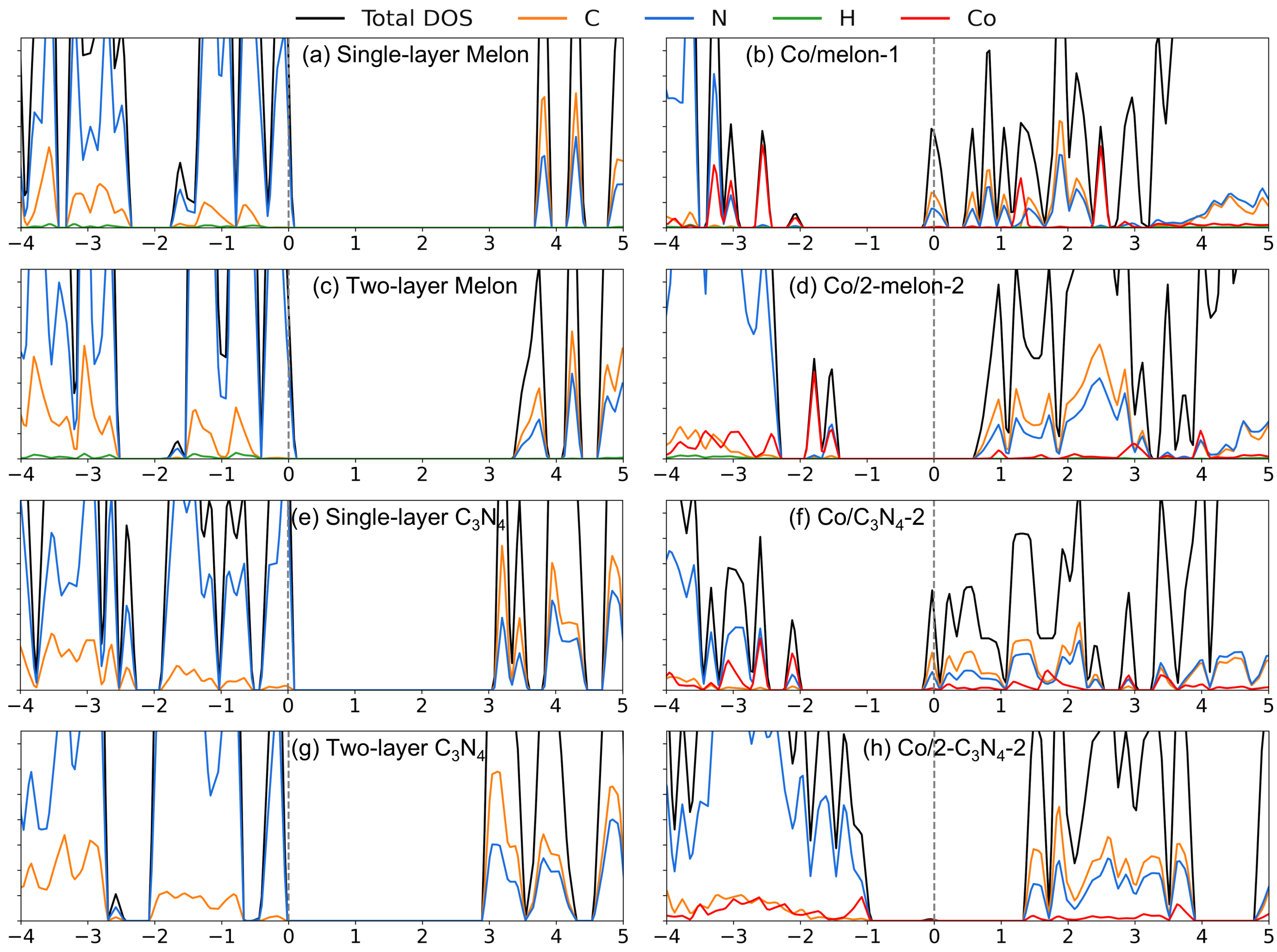

Figure S7: Density of states (DOS) plots for (a) single-layer melon, (c) two-layer melon, (e) single-layer g-C_3N_4, (g) two-layer g-C_3N_4, and (b), (d), (f) and (h) selected Co-bound polymeric carbon nitride structures. These Co/polymeric carbon nitrides are the most stable of those we modeled. The energies are relative to the Fermi level, which has been shifted to 0 eV and indicated by a dashed vertical line. Due to smearing of the electronic states, the Fermi level appears within the valence band, even though the HOMO energy level is below the Fermi level.

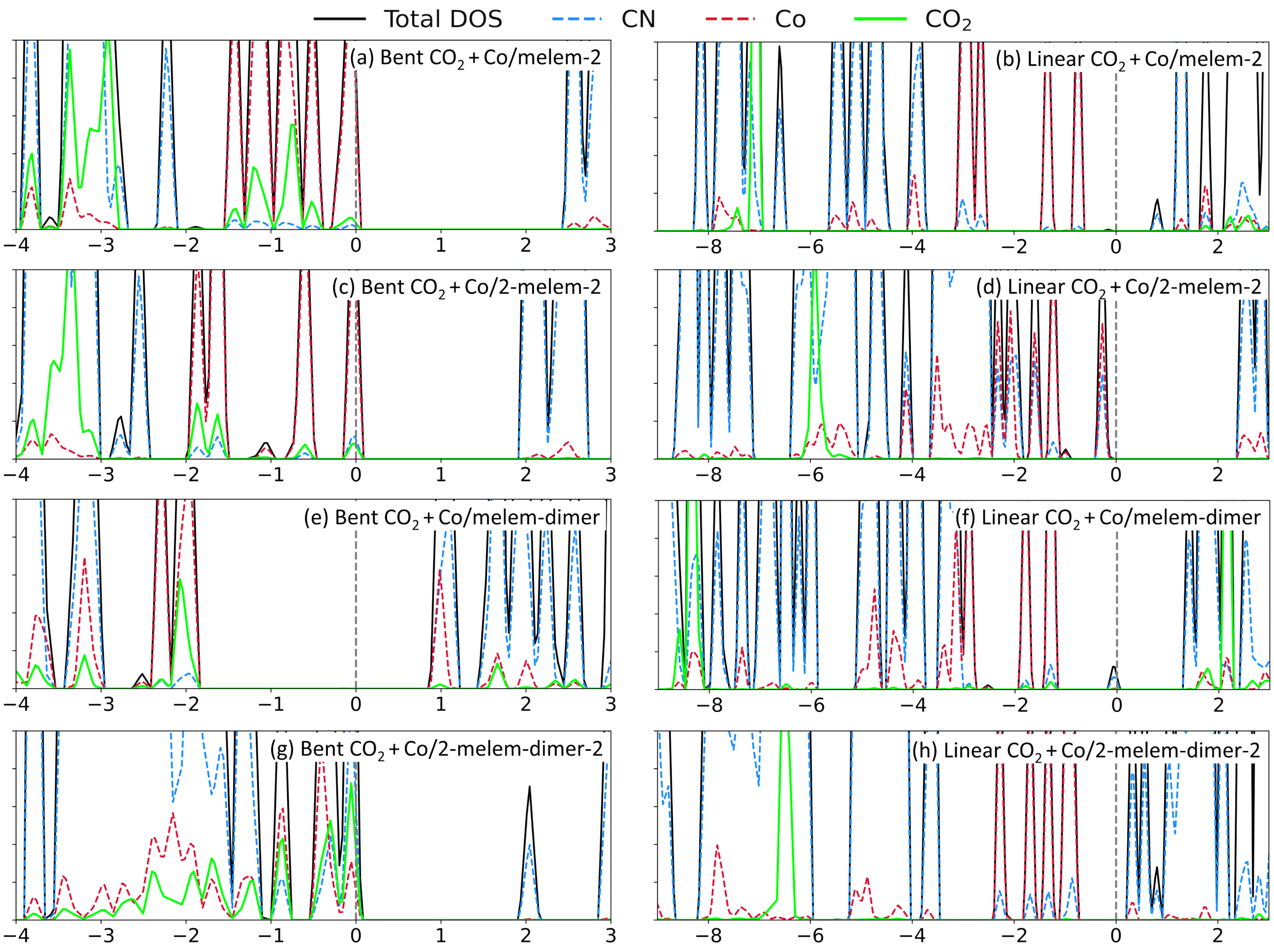

Figure S8: Density of states (DOS) plots for both bent and linear CO_2 on Co/melem-2, Co/2-melem-2, Co/melem dimer, and Co/2-melem-dimer-2. These complexes are the most stable of those we modeled. The energies are relative to the Fermi level, which has been shifted to 0 eV and indicated by a dashed vertical line. Due to smearing of the electronic states, the Fermi level appears within the valence band, even though the HOMO energy level is below the Fermi level.

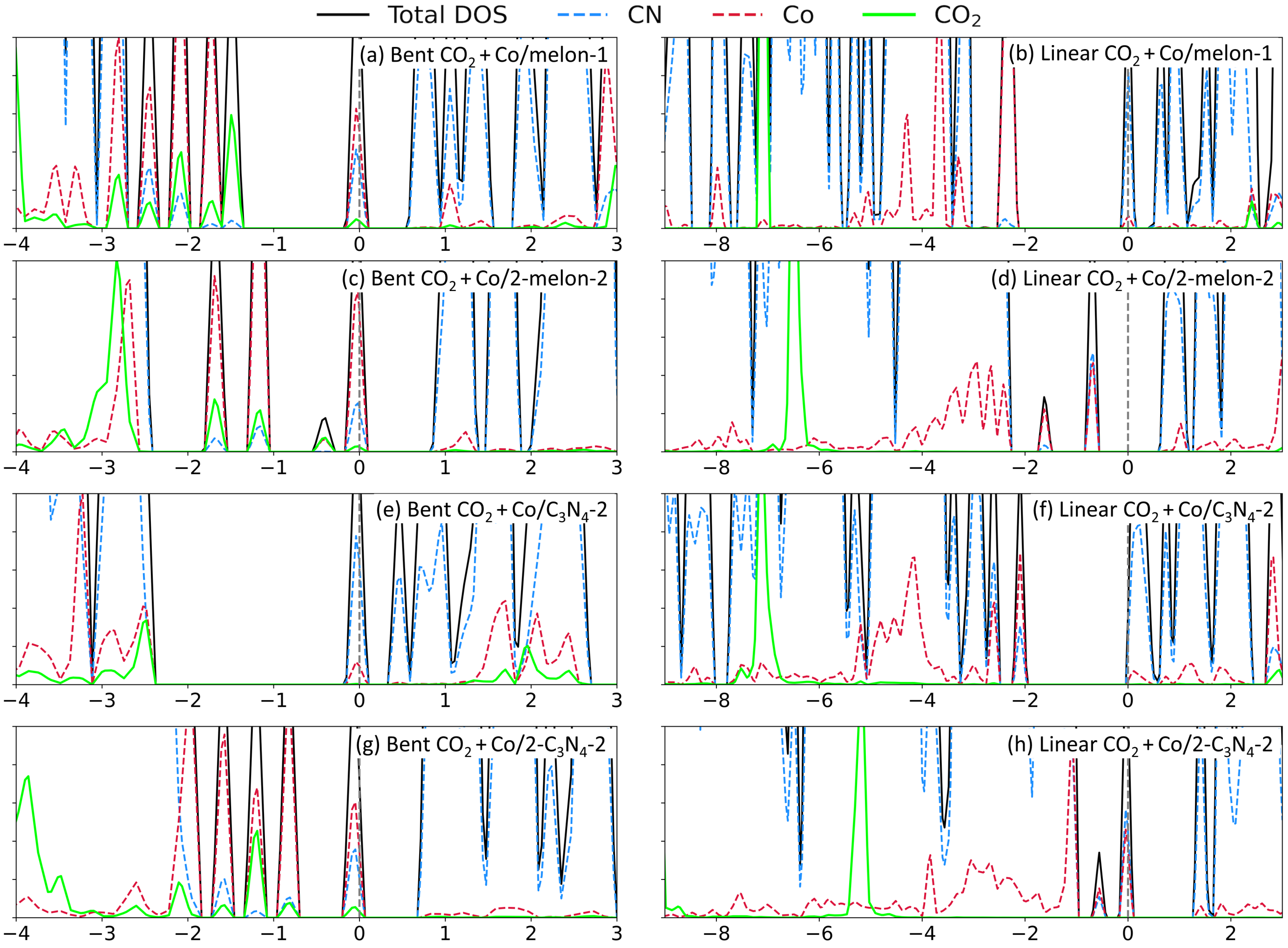

Figure S9: Density of states (DOS) plots for both bent and linear CO_2 on Co/melon-1, Co/2-melon-2, Co/C_3N_4-2, and Co/2-C_3N_4-2. These complexes are the most stable of those we modeled. The energies are relative to the Fermi level, which has been shifted to 0 eV and indicated by a dashed vertical line. Due to smearing of the electronic states, the Fermi level appears within the valence band, even though the HOMO energy level is below the Fermi level.

7 CO_2 Adsorption to Co/Carbon Nitrides

Figure S10 shows both linear and bent CO_2 adsorbed to the most stable Co/carbon nitride complexes. Adsorption energies are presented in Table 3 of the main text.

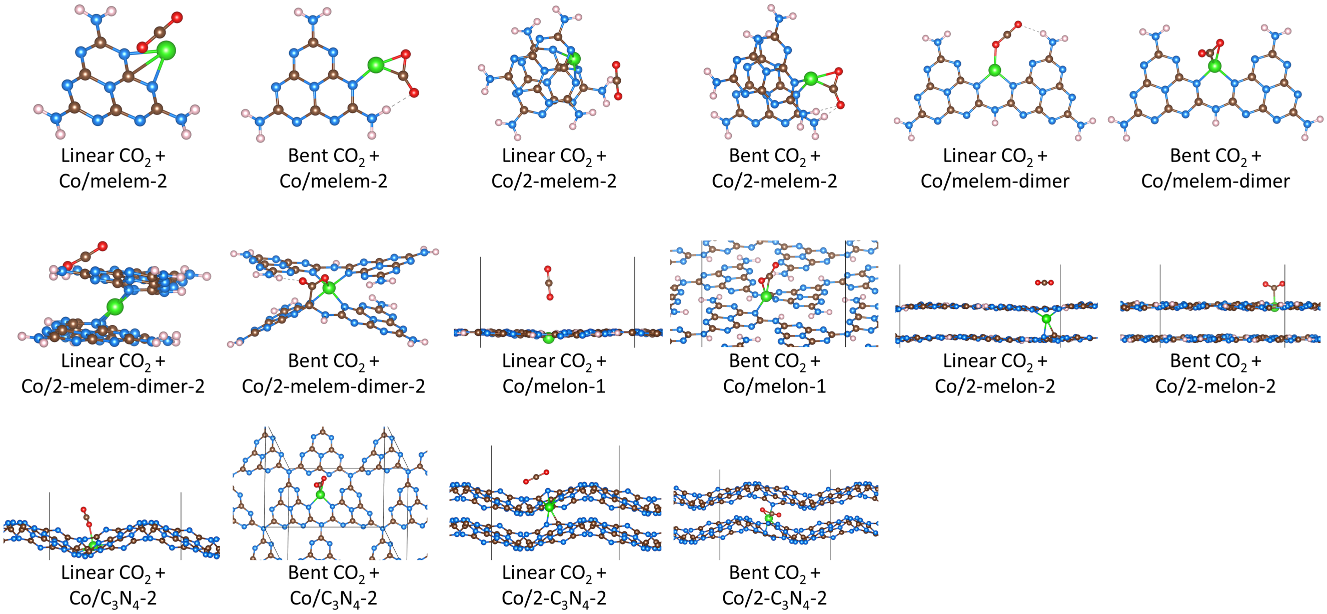

Figure S10: Stable linear and bent CO_2 on the most stable Co/carbon nitride structures. Brown spheres represent carbon atoms, blue spheres represent nitrogen atoms, green spheres represent cobalt atoms, red spheres represent oxygen atoms, and pink spheres represent hydrogen atoms.

References

[1] S. T. Melissen, T. Le Bahers, P. Sautet and S. N. Steinmann, *Physical Chemistry Chemical Physics*, 2021, **23**, 2853–2859.

[2] F. Fina, S. K. Callear, G. M. Carins and J. T. S. Irvine, *Chemistry of Materials*, 2015, **27**, 2612–2618.

[3] B. Lotsch, M. Döblinger, J. Sehnert, L. Seyfarth, J. Senker, O. Oeckler and W. Schnick, *Chemistry – A European Journal*, 2007, **13**, 4969–4980.

[4] S. T. Melissen, S. N. Steinmann, T. Le Bahers and P. Sautet, *Journal of Physical Chemistry C*, 2016, **120**, 24542–24550.

[5] F. Yu, T. Huo, Q. Deng, G. Wang, Y. Xia, H. Li and W. Hou, *Chemical Science*, 2022, **13**, 754–762.

[6] J. Lin, L. Jiang, W. Tian, Y. Yang, X. Duan, Y. Jiao, H. Zhang and S. Wang, *Journal of Materials Chemistry A*, 2023, **11**, 13653–13664.

[7] T. Botari, W. P. Huhn, V. W.-h. Lau, B. V. Lotsch and V. Blum, *Chemistry of Materials*, 2017, **29**, 4445–4453.

[8] C. Im, B. Kirchhoff, I. Krivtsov, D. Mitoraj, R. Beranek and T. Jacob, *Chemistry of Materials*, 2023, **35**, 1547–1559.

[9] V. W.-h. Lau, M. B. Mesch, V. Duppel, V. Blum, J. Senker and B. V. Lotsch, *Journal of the American Chemical Society*, 2015, **137**, 1064–1072.

[10] G. A. Meek, A. D. Baczewski, D. J. Little and B. G. Levine, *The Journal of Physical Chemistry C*, 2014, **118**, 4023–4032.

[11] H.-Z. Wu, L.-M. Liu and S.-J. Zhao, *Physical Chemistry Chemical Physics*, 2014, **16**, 3299.

[12] M. Zheng, H. Xu, Y. Li, K. Ding, Y. Zhang, C. Sun, W. Chen and W. Lin, *The Journal of Physical Chemistry C*, 2021, **125**, 13880–13888.

[13] J. Fu, L. Zhu, K. Jiang, K. Liu, Z. Wang, X. Qiu, H. Li, J. Hu, H. Pan, Y. R. Lu, T. S. Chan and M. Liu, *Chemical Engineering Journal*, 2021, **415**, 128982.

[14] K. Homlamai, T. Maihom, S. Choomwattana, M. Sawangphruk and J. Limtrakul, *Applied Surface Science*, 2020, **499**, 143928.

[15] D. Ghosh, G. Periyasamy, B. Pandey and S. K. Pati, *J. Mater. Chem. C*, 2014, **2**, 7943–7951.

[16] Z. Chen, J. Zhao, C. R. Cabrera and Z. Chen, *Small Methods*, 2019, **3**, 1800368.

[17] Q. Gao, X. Zhuang, S. Hu and Z. Hu, *The Journal of Physical Chemistry C*, 2020, **124**, 4644–4651.

[18] C. Ao, B. Feng, S. Qian, L. Wang, W. Zhao, Y. Zhai and L. Zhang, *Journal of CO2 Utilization*, 2020, **36**, 116–123.

[19] S. An, G. Zhang, T. Wang, W. Zhang, K. Li, C. Song, J. T. Miller, S. Miao, J. Wang and X. Guo, *ACS Nano*, 2018, **12**, 9441–9450.

[20] J. Shen, C. Luo, S. Qiao, Y. Chen, Y. Tang, J. Xu, K. Fu, D. Yuan, H. Tang, H. Zhang and C. Liu, *ACS Catalysis*, 2023, **13**, 6280–6288.

[21] C. Guo, T. Zhang, X. Deng, X. Liang, W. Guo, X. Lu and C. L. Wu, *ChemSusChem*, 2019, **12**, 5126–5132.

[22] E. Moharramzadeh Goliaei, *Langmuir*, 2024, **40**, 7871–7882.

[23] X. Tang, W. Shen, D. Li, B. Li, Y. Wang, X. Song, Z. Zhu and P. Huo, *Journal of Alloys and Compounds*, 2023, **954**, 170044.

[24] S. Liu, Y. Li, Y. Zhang and W. Lin, *Physical Chemistry Chemical Physics*, 2023, **25**, 9901–9908.
